# The JmjN domain as a dimerization interface and a targeted inhibitor of KDM4 demethylase activity

**DOI:** 10.18632/oncotarget.24717

**Published:** 2018-03-30

**Authors:** May Levin, Michal Stark, Yehuda G. Assaraf

**Affiliations:** ^1^ The Fred Wyszkowski Cancer Research Laboratory, Department of Biology, Technion-Israel Institute of Technology, Haifa 3200003, Israel

**Keywords:** KDM4 subfamily, JmjN domain, dimerization, histone demethylases, chromatin

## Abstract

Histone methylation is regulated to shape the epigenome by modulating DNA compaction, thus playing central roles in fundamental chromatin-based processes including transcriptional regulation, DNA repair and cell proliferation. Histone methylation is erased by demethylases including the well-established KDM4 subfamily members, however, little is known about their dimerization capacity and its impact on their demethylase activity. Using the powerful bimolecular fluorescence complementation technique, we herein show the *in situ* formation of human KDM4A and KDM4C homodimers and heterodimers in nuclei of live transfectant cells and evaluate their H3K9me3 demethylation activity. Using size exclusion HPLC as well as Western blot analysis, we show that endogenous KDM4C undergoes dimerization under physiological conditions. Importantly, we identify the JmjN domain as the KDM4C dimerization interface and pin-point specific charged residues therein to be essential for this dimerization. We further demonstrate that KDM4A/C dimerization is absolutely required for their demethylase activity which was abolished by the expression of free JmjN peptides. In contrast, KDM4B does not dimerize and functions as a monomer, and hence was not affected by free JmjN expression. KDM4 proteins are overexpressed in numerous malignancies and their pharmacological inhibition or depletion in cancer cells was shown to impair tumor cell proliferation, invasion and metastasis. Thus, the KDM4 dimer-interactome emerging from the present study bears potential implications for cancer therapeutics via selective inhibition of KDM4A/C demethylase activity using JmjN-based peptidomimetics.

## INTRODUCTION

Histone methylation is a key post-translational modification modulating chromatin compaction and consequently regulating gene expression, alternative splicing and other nucleic acid-associated processes [1–5]. Histones can be methylated on specific lysine residues by different lysine methyl transferases (KMTs), resulting in mono-, di- or trimethylated states [6]. This methylation is erased by specific histone lysine demethylases (KDMs) [6]. There are two known molecular mechanisms of lysine demethylation; the first is an FAD-dependent amine oxidation, catalyzed by the lysine specific demethylases LSD1/2 [7, 8]. The second involves a dioxygenase reaction which requires Fe^2 +^, O_2_, and α-ketoglutarate cofactors, and is catalyzed by Jumonji C domain-containing (JMJD) proteins [9, 10]. A major subfamily of the latter proteins is JMJD2, also known as KDM4, the members of which include KDM4A-E that target di- and trimethylated H3K9, H3K36 and H3K56 as well as trimethylated H1.4K26 [11–14]. While methylation of H3K36 is usually considered an epigenetic activation mark [11, 12], H3K9 di- (H3K9me2) and trimethylation (H3K9me3) and H1.4K26me3 are commonly known as repression marks [4, 11]. Moreover, H3K9 methylation is involved in chromatin condensation necessary for cell division [15] and mediates heterochromatin formation during DNA double strand break response [16].

KDM4 proteins share a very similar structure, as all contain N-terminal JmjN and JmjC domains, while KDM4A-C also harbor a double plant homodomain (PHD) and a double Tudor domain [17]. The PHD and Tudor domains possess histone reader functions facilitating the recruitment and binding of KDM4 proteins to specific chromatin marks [11]. JmjC is the catalytic domain [9] and is the most studied determinant of this group of demethylases. The function of the JmjN domain is still unknown, however it was found to be essential for their demethylase activity [18–21].

While certain lysine demethylases are either up- or downregulated in different malignancies [22], KDM4 family members are consistently overexpressed in numerous cancers [23] in which they were shown to drive tumorigenesis, invasion and metastasis [22, 24–32]. Consistently, pharmacological inhibition or silencing of KDM4 demethylases leads to attenuation of cell growth [33–35]. Thus, these important enzymes recently became an attractive drug target for cancer therapeutics [36–38]. In order to enable rational design of such anticancer compounds, it is necessary to enhance our understanding of the mechanisms underlying regulation of KDM4-dependent demethylation and how this catalytic activity is regulated in the context of the full protein. In this respect, a recent study revealed the existence of protein-protein interactions within the KDM4 family. Shin and Janknecht demonstrated co-immunoprecipitation of KDM4A and KDM4C both with themselves and with each other [20]. They further showed that amino acids (aa) 1–300 in KDM4A were required for these interactions, while deletion of aa 1–60 from KDM4A or 1–70 from KDM4C (i.e. JmjN domain) abolished their H3K9me3 and H3K36me2/3 demethylation activity. Based on these findings, we herein hypothesized that dimerization of specific members of the KDM4 family, via their JmjN domains, is essential for their demethylation activity. To explore the validity of this hypothesis, we applied the bimolecular fluorescence complementation (BiFC) technique on both the full-length KDM4 proteins as well as on isolated KDM4 domains. This powerful technique can follow subcellular *in situ* protein dimerization in live cells along with its catalytic activity. Immunofluorescence microscopy and Western blot (WB) analyses revealed the formation of KDM4A and KDM4C homo- and heterodimers, whereas KDM4B did not display any form of dimerization. We further identified the JmjN domain as the dimerization interface of KDM4C and pin-pointed specific charged residues as essential for this dimerization. In addition, we provide several lines of experimental evidence indicating that dimerization of KDM4A and KDM4C is absolutely required for their catalytic activity. Finally, we show that expression of the JmjN peptide abolished the demethylase activity of exogenous KDM4A and KDM4C, whereas KDM4B retained its activity. These findings identify the JmjN as a possible druggable target in the KDM4 family as well as a potential inhibitor for selective cancer therapeutics. Collectively, our findings suggest an emerging dimerization interactome of KDM4 family members, hence bearing important implications for substrate specificity and various key biological functions of this central family of demethylases.

## RESULTS

### Expression, localization and activity of YFP-conjugated KDM4A-C

**Figure 1:**
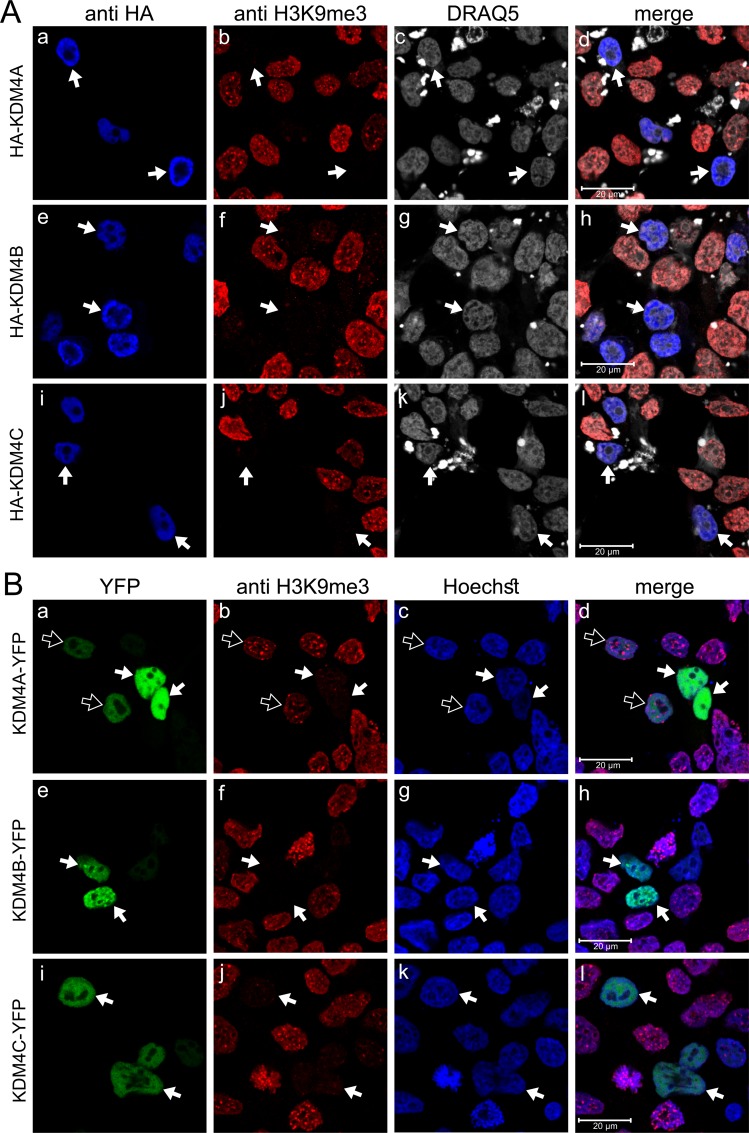
IF microscopy images displaying subcellular localization and activity of exogenous KDM4A-C proteins HEK293 cells were transfected with expression vectors harboring the KDM4A-C proteins with either an N-terminal HA tag (**A**) or C-terminal YFP tag (**B**) and visualized by IF microscopy. Blue fluorescence represents either anti-HA staining (A) or the DNA dye Hoechst 33342 (B); Red fluorescence represents H3K9me3, which allows monitoring demethylation activity; Green fluorescence denotes the YFP signal; White fluorescence represents the DNA dye DRAQ5; White arrows indicate specific cells with expression of the exogenous enzymes, which are localized at the nuclei and display demethylation activity; Outlined arrows point to cells with expression of inactive KDM4A-YFP. Cells were analyzed by a scanning confocal microscope at a ×63 magnification. All fields are representative of at least three independent experiments.

To explore the *in situ* dimerization capacity of KDM4A-C using the established BiFC technique, we first generated YFP-tagged full-length KDM4A-C constructs. The expression vectors were transiently introduced into HEK293 cells, and immunofluorescence (IF) microscopy was performed to confirm that the C-terminal conjugation to the large YFP protein neither interfered with KDM4A-C expression and nuclear localization nor with their demethylation activity (Figure 1). We used the original HA-tagged KDM4 expression vectors as controls, since they were previously shown to retain both proper nuclear localization and demethylase activity [39]. An anti-HA antibody (blue fluorescence) was used to validate their expression (Figure 1A, left panel), while their demethylation activity was evaluated by a reduction in the H3K9me3 red fluorescence (Figure 1A, b, f and j). For the YFP-conjugated enzymes, YFP green fluorescence confirmed their intact fold and expression (Figure 1B, left panel), and nuclear localization was verified by the overlapping green fluorescence of YFP and the blue fluorescence of the DNA dye Hoechst 33342 (Figure 1B, d, h and l). All three YFP-conjugated KDM4 proteins were highly expressed and localized solely in nuclei (Figure 1B, right panel). However, while KDM4B and KDM4C retained full demethylase activity, as demonstrated by the complete loss of H3K9me3 staining in YFP positive cells (Figure 1B, indicated by white arrows), KDM4A-YFP displayed reduced H3K9me3 demethylation activity compared to the original HA-KDM4A (Figure 1, compare 1B-b to 1A-b); very high KDM4A-YFP expression levels allowed the demethylation of H3K9me3, whereas low to moderate expression levels were not sufficient to exert detectable demethylase activity (Figure 1B-b, compare white-filled arrows to outlined arrows), suggesting that the large YFP tag interferes with the demethylase activity of KDM4A.

### KDM4A and KDM4C, but not KDM4B, form homo- and heterodimers

**Figure 2:**
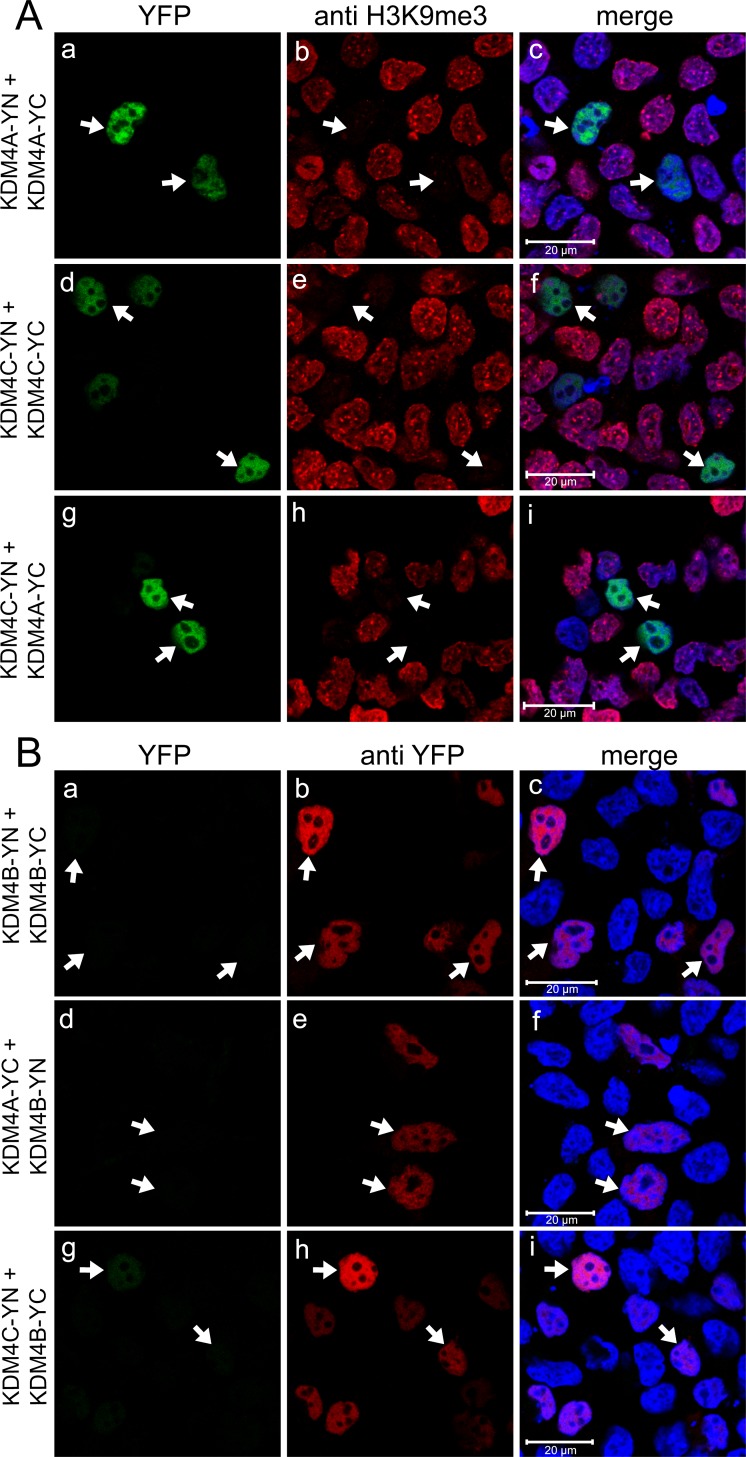
Expression and dimerization of KDM4A-C with a C-terminal YN or YC tag IF microscopy in HEK293 cells displaying homo- and heterodimerization of KDM4A and 4C (**A**), or expression of KDM4B with no detectable formation of homo- and heterodimers (**B**). Green fluorescence represents YFP that was reconstituted by the refolding of the non-fluorescent YN and YC halves, red fluorescence represents H3K9me3 (A) or anti-YFP staining (B), whereas blue fluorescence denotes the DNA dye Hoechst 33342. Arrows point to specific cells with formation of KDM4A/C homo- and heterodimers which display demethylation activity (A), or to cells displaying anti-YFP staining but lacking YFP fluorescence (B). Cells were visualized by a scanning confocal microscope at a ×63 magnification. All fields are representative of at least three independent experiments.

To initially assess possible dimerization of KDM4 family members, YC- and YN-tagged KDM4A-C were expressed in HEK293 cells and visualized by live cell imaging (Supplementary Figure 1). YFP fluorescence was detected following proper reconstitution of its two halves, thus confirming the formation of KDM4A and KDM4C homodimers, as well as KDM4A-KDM4C heterodimers (Supplementary Figure 1A, 1E and 1I, respectively). However, transfections with KDM4B resulted in very weak YFP signals, indicating poor homodimerization capacity of KDM4B as well as heterodimerization of KDM4B with KDM4A/C (Supplementary Figure 1C, 1G and 1K, respectively). KDM4A and KDM4C homo- and heterodimers were then visualized by IF assays to assess their demethylase activity (Figure 2A). H3K9me3 fluorescence was erased in the YFP-positive nuclei of transfectant cells (Figure 2A, middle panel, arrows), confirming the demethylase activity of exogenous KDM4A/C YN and YC conjugates. Notably, the YN and YC tags did not impair the demethylase activity of KDM4A, suggesting that the relatively small YN and YC tags did not exert a deleterious effect as was observed with the relatively large YFP tag (Figure 2A-b compared to Figure 1B-b).

**Figure 3:**
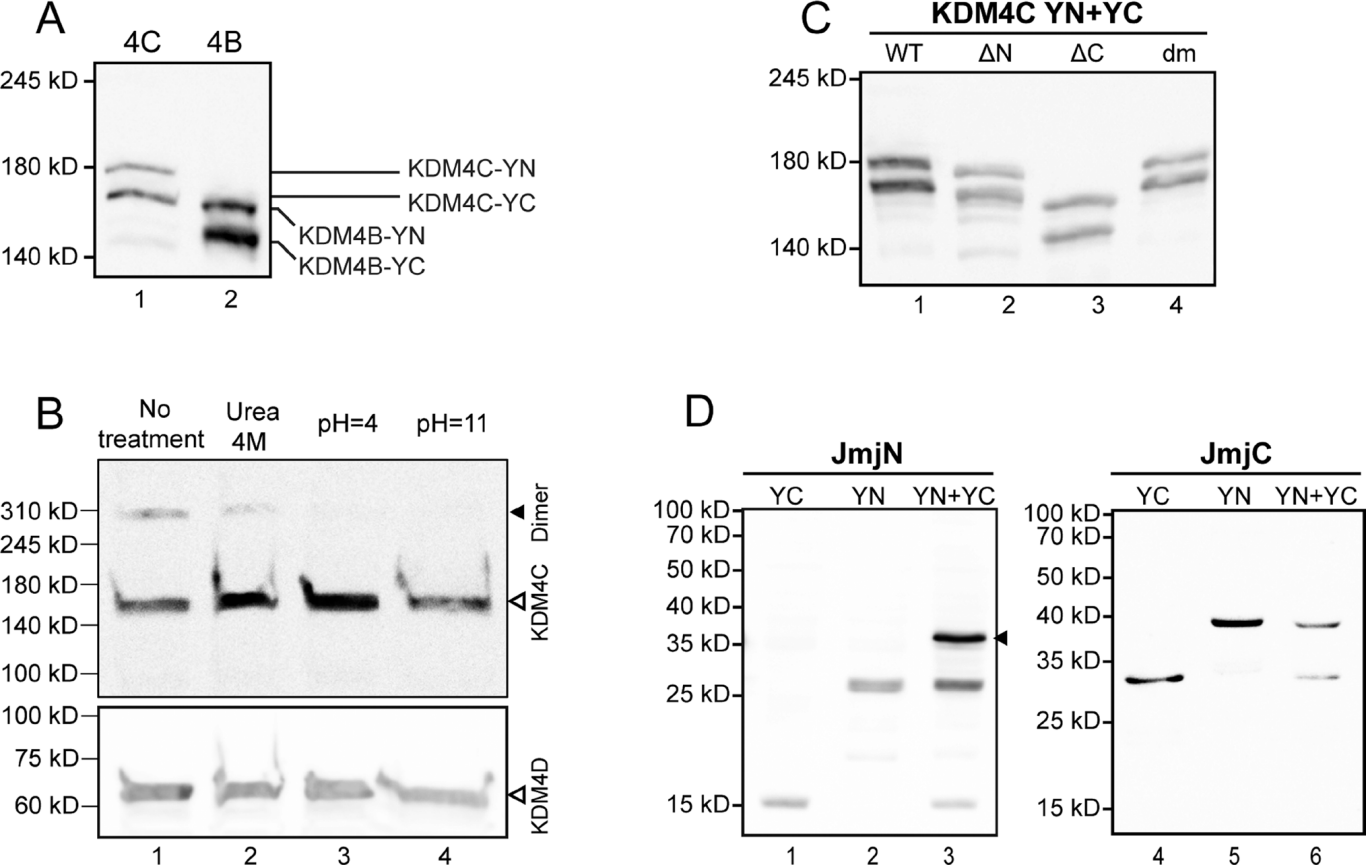
Evaluation of protein expression by Western blot analyses The expression of exogenous YN- and YC-tagged proteins was evaluated following transfections of HEK293 cells, while expression and dimerization of the endogenous KDM4C was evaluated in K562 cells, as described in the Methods section. (**A**) Expression of YN- and YC-tagged KDM4C and KDM4B in nuclear extracts detected by anti-YFP specific antibody; (**B**) Expression and dimerization of endogenous nuclear KDM4C, treated with either 4 M urea, pH = 4 or pH = 11 buffers, and visualized by a KDM4C-specific antibody (upper panel). The size of KDM4C dimers is indicated by a solid arrowhead. The membrane was stripped and reprobed with an anti-KDM4D antibody as a loading control (bottom panel); (**C**) Nuclear expression of the YN- and YC-tagged WT and mutant KDM4C lacking the JmjN domain (ΔN) or the JmjC domain (ΔC), as well as the double mutant harboring p.E24A/E25A + p.H42A/R43A (dm), visualized by a KDM4C-specific antibody; (**D**) Expression and dimerization capacity of the individual YN- and YC-tagged JmjN and JmjC domains in total cell extracts, detected by an anti-YFP antibody. The JmjN homodimer is indicated by an arrowhead. The results are representative of at least three independent experiments.

**Figure 4:**
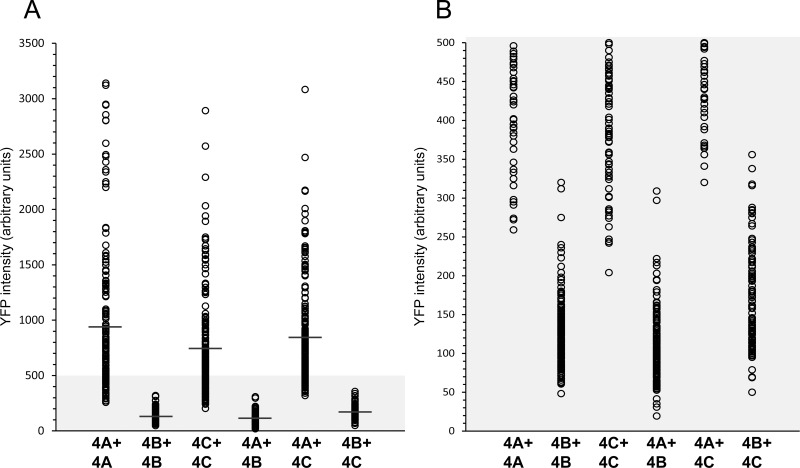
Quantitative image analysis of the levels of KDM4A-C homo- and heterodimerization HEK293 cells were co-transfected with each of the YN- and YC-tagged KDM4A-C pairs (i.e. KDM4A-YC + KDM4A-YN, KDM4B-YC + KDM4B-YN, KDM4C-YC + KDM4C-YN, KDM4A-YC + KDM4B-YN, KDM4A-YC + KDM4C-YN or KDM4B-YC + KDM4C-YN), and IF microscopy was performed. An anti-YFP specific antibody was used to mark transfected cells while the reconstitution of the YFP fluorescence represented the level of dimerization. Cells were visualized by a scanning confocal microscope at a ×63 magnification, and images were analyzed using the *Imaris* image analysis software. Each circle represents the mean YFP fluorescence intensity in a positively transfected cell. The average YFP fluorescence of each group is marked by a horizontal line. An expanded view of the lower section of the Y axis (YFP intensity ≤500) is depicted in panel B. Statistical parameters are detailed in Table 1. Any comparison between a pair containing KDM4B to a pair consisting solely of KDM4A/C had a *P*-value ≤ 7.4 × 10^−32^.

To confirm that the individual YC- and YN conjugates of KDM4B did not dimerize, and that this result is not an artifact of impaired protein expression, we verified their actual expression by IF microscopy and WB analyses using an antibody against YFP (Figure 2B and Figure 3A, respectively). In the IF assays, comparable staining levels of the anti-YFP antibody (red fluorescence) were observed in cells transfected with both KDM4B conjugates (i.e. KDM4B-YN and KDM4B-YC), as in cells transfected with KDM4B along with KDM4A/C (i.e. KDM4B-YN with KDM4A-YC or KDM4B-YC with KDM4C-YN) (Figure 2B, middle panel). The comparable levels of anti-YFP staining suggest similar expression levels of KDM4B and KDM4A/C, yet no YFP fluorescence was observed (Figure 2B, left panel). Furthermore, both KDM4B YN and -YC monomers were detected upon WB analysis at higher levels than that of KDM4C (Figure 3A, compare lane 2 to lane 1). These results indicate that KDM4B does not undergo efficient homodimerization or heterodimerization with KDM4A and KDM4C. In order to compare the difference in the dimerization capacity of KDM4A/C and KDM4B, we performed IF microscopy followed by quantitative image analysis using the *Imaris* software as detailed under Materials and Methods. HEK293 cells were co-transfected with each of the YN- and YC-tagged KDM4A-C pairs and immunostained with the anti-YFP antibody. The quantitative analysis was performed on >100 positively-transfected cells for each transfection, in which the mean intensity of the reconstituted YFP fluorescence was determined (Figure 4). YFP fluorescence values for each transfection are depicted in Table 1. This analysis revealed that every KDM4B-containing transfection (i.e. 4B + 4B, 4B + 4A, 4B + 4C) had very low YFP fluorescence, such as that the highest fluorescence recorded for KDM4B was comparable to the lowest fluorescence obtained for KDM4A and KDM4C (Figure 4B and Table 1). Any combination of KDM4A/C displayed a ~6-fold higher average fluorescence than the KDM4B-containing combinations (Table 1, *P*-values ≤ 7.4 × 10^−32^), indicating that KDM4B has a very low dimerization capacity within the KDM4 subfamily.

**Table 1:** Dimerization analysis

Transfection	*n* (cells)	YFP fluorescence (arbitrary units)			
		Min	Max	Average	Median
**KDM 4A + 4A**	163	259	3141	**943**	**726**
**KDM 4B + 4B**	170	48	320	**129**	**120**
**KDM 4C + 4C**	213	204	2892	**741**	**628**
**KDM 4A + 4B**	135	19	309	**110**	**104**
**KDM 4A + 4C**	217	320	3083	**845**	**717**
**KDM 4B + 4C**	114	50	356	**170**	**146**

### Endogenous KDM4C from K562 cells exists in high molecular weight complexes, consistent with dimers and tetramers

**Figure 5:**
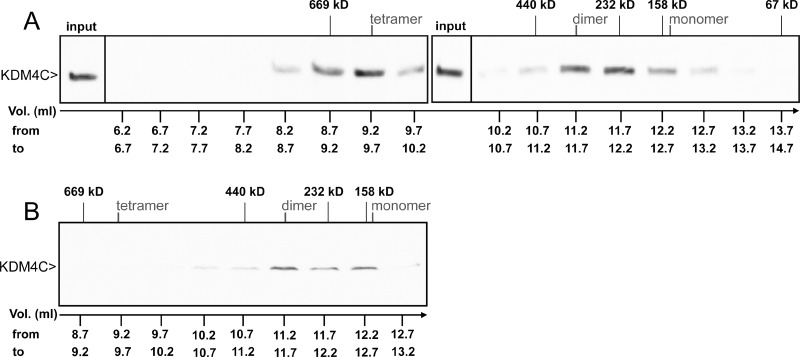
WB analysis of endogenous KDM4C from size exclusion HPLC fractions K562 cells were harvested for NP (**A**) or CP (**B**) extractions. Samples were loaded on a gel filtration Superdex 200 10/300 GL column, eluted in 0.5 ml fractions and analyzed by WB with an anti-KDM4C antibody, in which a sample of the NP input was used as a positive control. Size markers represent the elution volumes of Thyroglobulin (669 kD), Ferritin (440 kD), Catalase (232 kD), Aldolase (158 kD) and BSA (67 kD). The fraction volume (Vol.) is indicated at the bottom of each lane, while the expected elution volumes of KDM4C monomers, dimers and tetramers are marked at the top.

The results above enabled us to visualize the interactions between exogenous YN- and YC-tagged KDM4A/C monomers. In order to assess the physiological relevance of said interactions, we aimed to determine whether or not the endogenous KDM4C undergoes dimerization. To this end, we used the human chronic myeloid leukemia cell line K562, which displays higher levels of endogenous KDM4C in comparison to the non-malignant HEK293 cell line. Initial experiments included WB analyses on nuclear protein (NP) extracts isolated from K562 cells, using a monoclonal anti-KDM4C antibody (Figure 3B). Two discrete bands were apparent: one consistent with the molecular weight (MW) of a KDM4C monomer (~150 kD), and another band with double the MW of the latter (~300 kD), consistent with a dimer (Figure 3B, lane 1). In order to verify that the higher MW band is not an artifact of an unspecific antibody, we treated the protein extract with known biochemical conditions that could dissociate protein dimers. It is apparent in Figure 3B, that the intensity of the higher MW band was reduced in the presence of 4 M urea, which weakens hydrophobic interactions (Figure 3B, lane 2), and was completely abolished at either low or high pH, both of which disrupt salt bridges (pH 4 and 11, respectively; Figure 3B, lanes 3–4). This was accompanied by an increase in monomer intensity following treatment with 4 M urea or acidic pH (Figure 3B, lanes 2–3). These results suggest that the higher MW band represents a protein complex containing KDM4C, possibly a KDM4C homodimer or a KDM4A/C heterodimer. Moreover, since the increase in the intensity of the monomer band exceeded the level of the original dimer, we further explored whether KDM4C endogenously exists in higher oligomeric structures, which would not appear in a WB analysis due to their size. Towards this end, we undertook size exclusion high pressure liquid chromatography (HPLC) of nuclear or cytosolic protein extracts from K562 cells. The eluted fractions were visualized by WB analysis following their acidification such that KDM4C would dissociate into monomers, which are more amenable to electroblotting (Figure 5). In the nuclear extract, KDM4C presented in fraction volumes that include the MW of KDM4C tetramers (9.7 ml, ~600 kD), dimers (11.2 ml, ~300 kD) and to a lesser extent monomers (12.6 ml, ~150 kD, Figure 5A). However, cytosolic KDM4C was only present in the fractions containing monomers and dimers (Figure 5B). These results lend support to the possible dimeric and tetrameric interactions of endogenous KDM4C under physiological conditions.

### Deletion of the JmjN domain from the full-length KDM4C abrogates dimer formation and H3K9me3 demethylation activity

**Figure 6:**
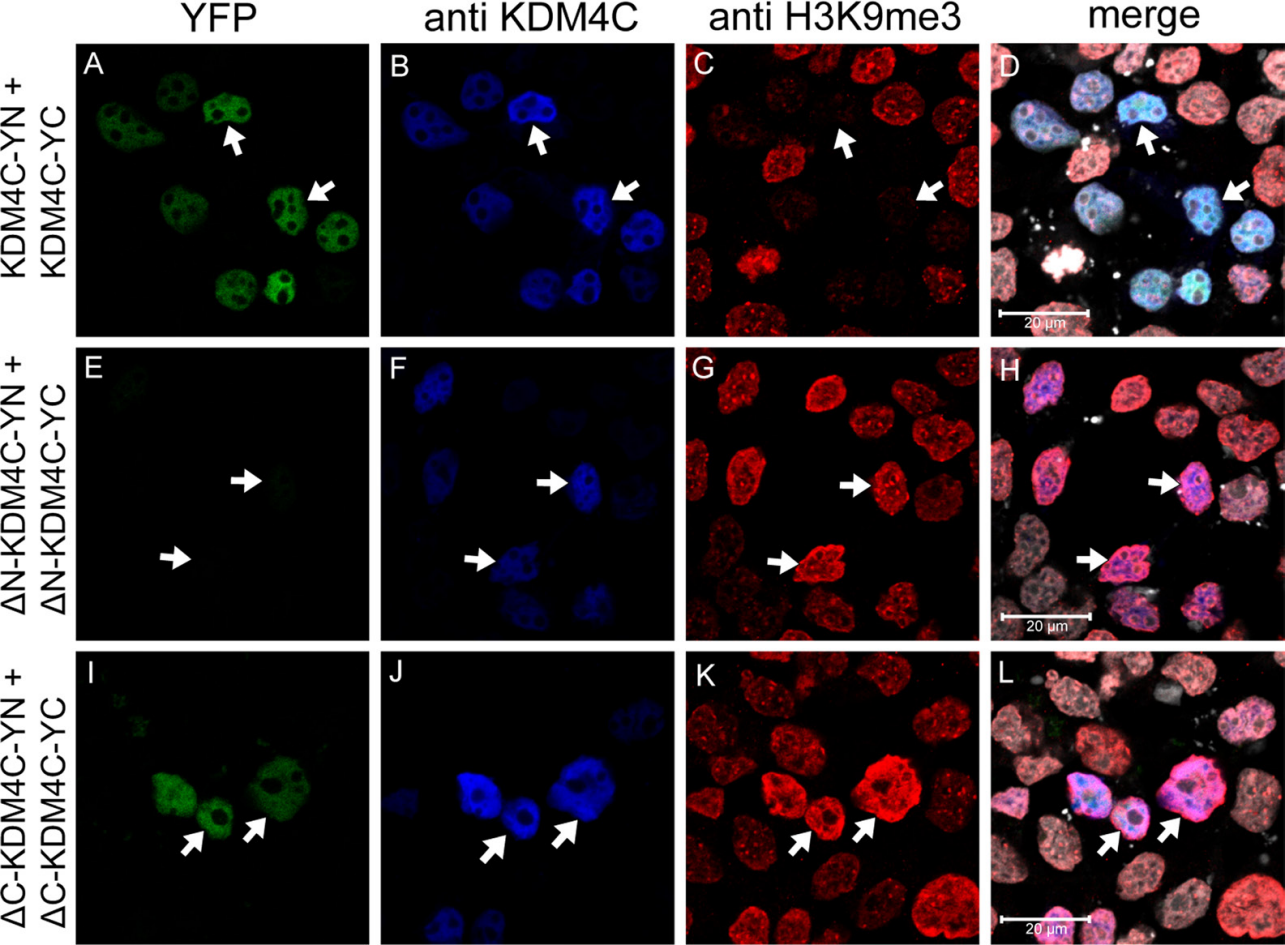
IF microscopy assessing the dimerization of KDM4C YN and YC monomers with deletion mutations HEK293 cells were transfected with expression vectors harboring WT KDM4C monomers, or deletion mutants; ΔN represents deletion of the JmjN domain, while ΔC represents deletion of the JmjC domain. Green fluorescence represents YFP that was reconstituted by the refolding of the YN and YC halves. Blue fluorescence represents anti-KDM4C staining and allows to identify transfected cells. Red fluorescence represents H3K9me3 which is reduced upon demethylation activity, and white fluorescence denotes the DNA dye DRAQ5. Arrows indicate specific cells with KDM4C overexpression; Cells were visualized by a scanning confocal microscope at a ×63 magnification. All fields are representative of at least three independent experiments.

After establishing that KDM4C dimerizes under physiological conditions, we aimed to determine whether or not the JmjN domain serves as its dimerization interface. Towards this end, KDM4C YN and YC conjugates lacking their JmjN domain (termed ΔN-KDM4C-YN/YC) were evaluated in comparison to the WT KDM4C, which served as a positive control in the following experiments: First, ΔN-KDM4C-YN and ΔN-KDM4C-YC were co-transfected into HEK293 cells and visualized by live cell imaging (Supplementary Figure 2A-c). No YFP fluorescence was detected, indicating that ΔN-KDM4C did not homodimerize. Second, IF microscopy was performed to verify ΔN-KDM4C expression using a KDM4C-specific antibody, and to test its demethylase activity with an anti-H3K9me3 antibody (Figure 6, blue and red fluorescence, respectively). ΔN-KDM4C monomers were properly detected and co-localized with the DNA dye DRAQ5 (Figure 6, right panel, white fluorescence); however, they did not display any YFP fluorescence, indicating that they did not dimerize (Figure 6, compare image E to image A). Furthermore, cells expressing ΔN-KDM4C had substantially higher levels of H3K9me3 when compared to untransfected cells in the same microscope field (Figure 6G, arrows), indicating that ΔN-KDM4C is devoid of H3K9me3 demethylation activity. It is possible that ΔN-KDM4C inhibits endogenous KDM4C by binding to H3K9me3 without exerting its demethylation activity, resulting in increased H3K9me3 levels. To show that not every deletion of a given KDM4C domain results in loss of dimerization capability, we constructed expression vectors harboring KDM4C YN/YC monomers which lack the JmjC domain (aa 144–310, ΔC-KDM4C-YN/YC) and tested their dimerization capacity using IF assays (Figure 6, bottom row). ΔC-KDM4C monomers were expressed at levels comparable to the WT- and ΔN-KDM4C monomers (Figure 6, compare images J, B and F, respectively) and displayed co-localization with H3K9me3 (Figure 6L). This implied that the ΔC-KDM4C monomers are properly sorted to the nucleus and that they are devoid of demethylase activity as previously shown [40]. In contrast to ΔN-KDM4C, ΔC-KDM4C monomers displayed YFP fluorescence demonstrating the retention of their homodimerization capacity (Figure 6I). The expression of both YN- and YC-monomers of the KDM4C deletion mutants was verified by WB analysis (Figure 3C). All monomers were expressed and visualized at the correct MW, though to a lesser degree. These findings support the conclusion that the JmjN domain is essential for the homodimerization of KDM4C. They also agree with previous reports that the JmjN domain is necessary for KDM4C demethylation activity [20]. Hence, we postulate that KDM4C dimerization is indispensable for its H3K9me3 demethylation activity.

### Individual JmjN but not JmjC domains, form homodimers

Once the essential role of the JmjN domain in KDM4C dimerization was demonstrated, we next aimed to study its sufficiency. To this end, we cloned the JmjN domain of KDM4A-C into the YN and YC vectors. First, the isolated YN- and YC-tagged JmjN monomers of KDM4A-C were co-expressed in HEK293 cells and visualized by live cell fluorescence imaging (Supplementary Figure 3). The JmjN domains of KDM4C and KDM4B exhibited YFP fluorescence, thus providing the first indication that independent JmjN peptides undergo dimerization (Supplementary Figure 3C and 3E). Moreover, YFP fluorescence was detected throughout the cell since JmjN has no nuclear localization sequence and has no chromatin-binding capability. Unlike the JmjN peptides from KDM4C and KDM4B which display stable expression, the JmjN domain of KDM4A appeared to aggregate (Supplementary Figure 3A, arrow), thus indicating that the isolated YN- or YC-tagged JmjN peptides from KDM4A are unstable and undergo degradation. We therefore did not pursue the JmjN domain from KDM4A any further.

To corroborate that the observed dimerization was driven by JmjN-JmjN interactions, rather than by YN-YC interactions, we aimed to examine a different peptide which does not dimerize under the same conditions. For this purpose, we studied the JmjC domain from KDM4C, which we found to be dispensable for its dimerization (Figure 6I). The JmjC YN and YC-tagged monomers were co-expressed in HEK293 cells and visualized by live cell fluorescence imaging (Supplementary Figure 3G). Expectedly, there was no dimerization of the JmjC monomers as indicated by the lack of YFP fluorescence (Supplementary Figure 3, compare image G to image E).

**Figure 7:**
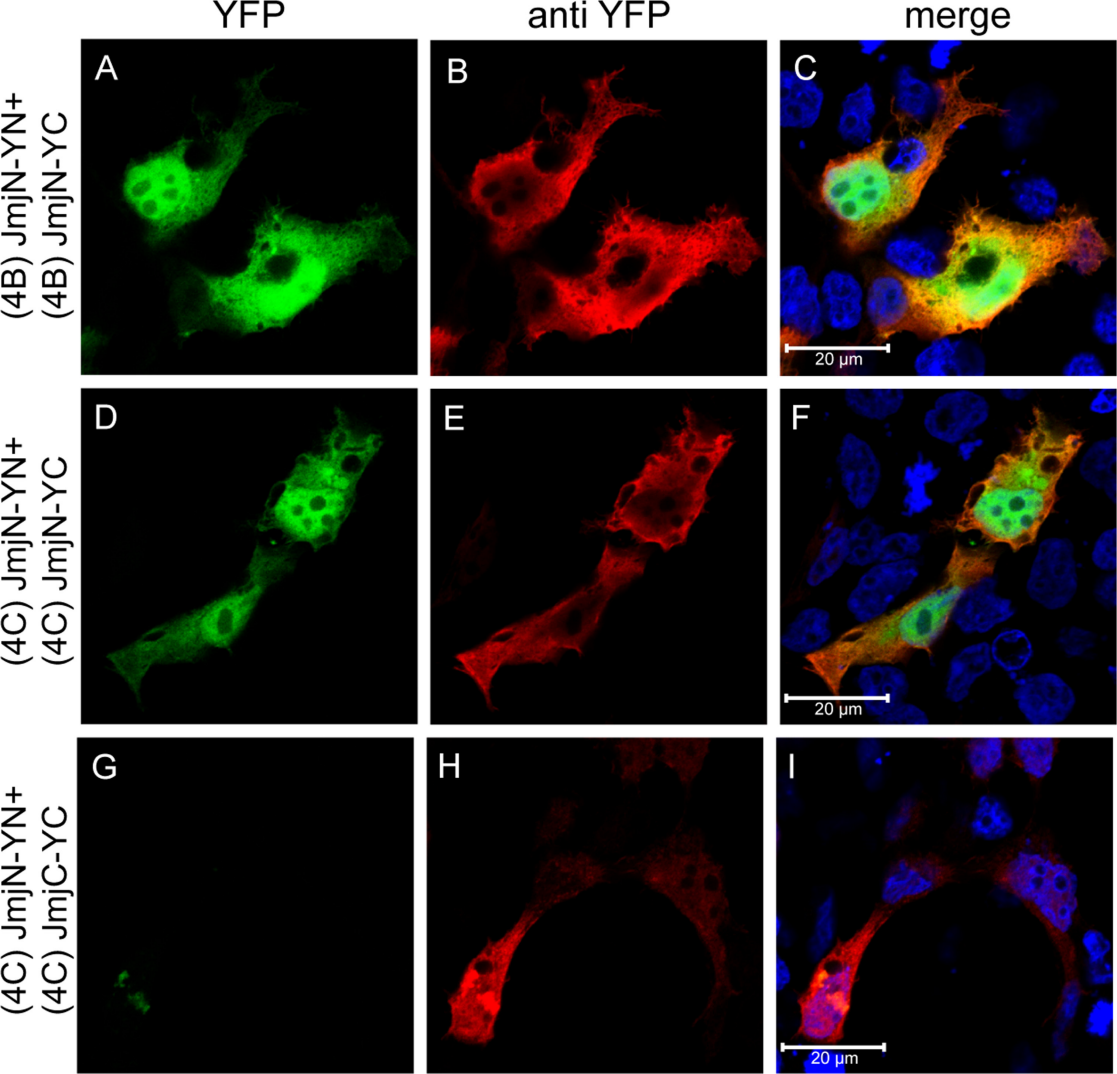
Expression and dimerization of individual JmjN and JmjC domains IF microscopy in HEK293 cells transfected with YN and YC-conjugated JmjN sequences from KDM4B/C, or JmjN and JmjC sequences from KDM4C. Green fluorescence represents YFP that was reconstituted by the refolding of the YN and YC halves. Red fluorescence represents anti-YFP staining which allows the detection of transfected cells, and blue fluorescence denotes the DNA dye Hoechst 33342. Cells were visualized by a scanning confocal microscope at a ×63 magnification. All fields are representative of at least three independent experiments.

To confirm that JmjN is not a sticky peptide that can interact with various irrelevant peptides, we performed IF microscopy in HEK293 cells co-transfected with YN- and YC-conjugated JmjN monomers from KDM4B/C or with JmjN-YN and JmjC-YC monomers using an antibody against YFP as a transfection marker (Figure 7, red fluorescence). The isolated JmjN from both enzymes displayed YFP fluorescence demonstrating dimerization, in concordance with the live cell imaging results (Figure 7A and 7D). The JmjN and JmjC peptides did not dimerize, as evidenced by the lack of YFP fluorescence (Figure 7G), thus emphasizing the precise nature of the JmjN-dependent homodimerization. Finally, the expression levels of the YN- and YC-conjugated JmjN and JmjC monomers were confirmed by WB analysis with an anti-YFP antibody (Figure 3D). While the YN- and YC-conjugated JmjN peptides were detected both as monomers and as a dimer (MW: 25, 15 and 40 kD, Figure 3D, lanes 1–3, respectively), JmjC YN and YC-tagged peptides were detected only as monomers (37 and 27 kD, Figure 3D, lanes 4–6, respectively). These findings confirmed that JmjN-JmjN interactions were the driving force for dimerization, and not a side effect of the YFP reconstitution.

### Charged/polar amino acids in the JmjN domain mediate KDM4C dimerization

**Figure 8:**
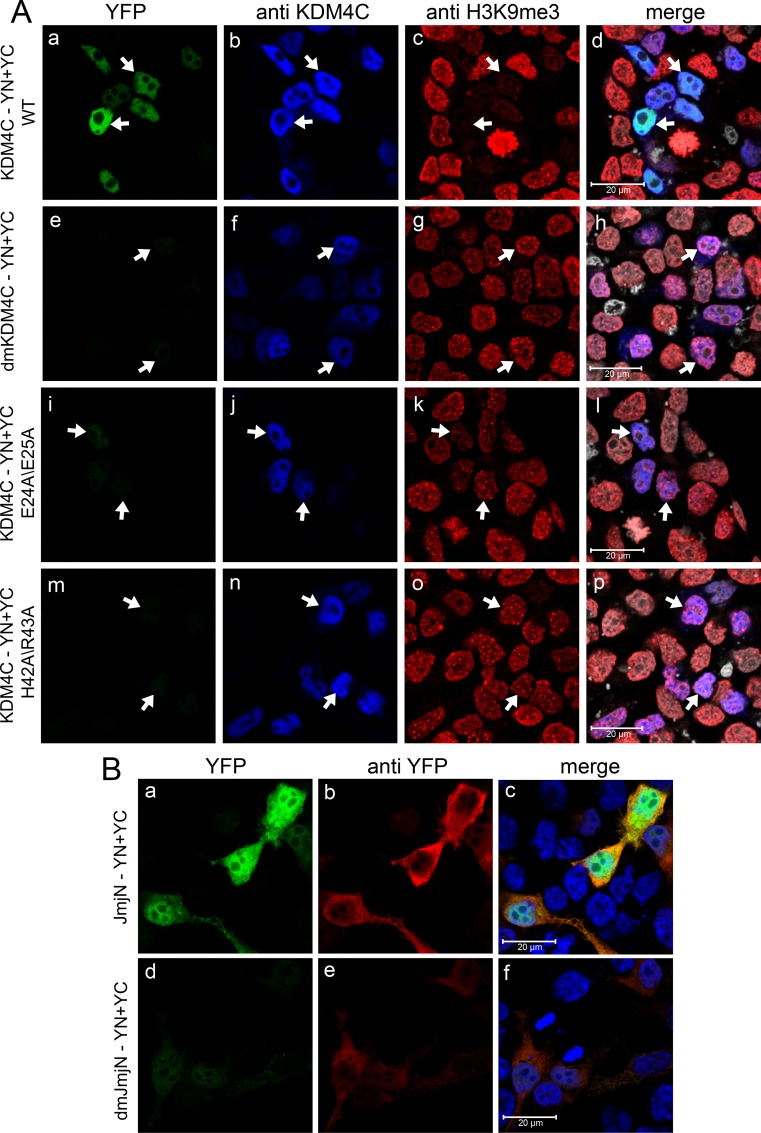
IF microscopy demonstrating the effect of point mutations within the JmjN sequence on dimerization and activity of KDM4C HEK293 cells were transfected with YN and YC-conjugated full length KDM4C (**A**) or independent JmjN (**B**) sequences harboring either p.E24A/E25A mutation, p.H42A/R43A mutation or both p.E24A/E25A + p.H42A/R43A mutations (termed dmKDM4C and dmJmjN). Green fluorescence represents YFP that was reconstituted by the refolding of the YN and YC halves. Blue fluorescence represents anti-KDM4C staining (A) or the DNA dye Hoechst 33342 (B). Red fluorescence represents H3K9me3 (A) or anti-YFP staining (B), whereas white fluorescence denotes the DNA dye DRAQ5. Arrows point out to cells with KDM4C overexpression. Cells were visualized by scanning confocal microscopy at a ×63 magnification. All fields are representative of at least three independent experiments.

To further characterize the JmjN-JmjN protein interactions, we sought to identify crucial residues within the domain that are required for this dimerization. To this end, we first aligned the peptide sequences of the JmjN domains from human KDM4A-C, and analyzed the conservation of the JmjN sequence in different organisms, using the NCBI *BLAST* tool (Supplementary Figure 4). We searched for specific amino acids with the following characteristics; 1) Residues located on one of the outward facing helices, i.e. α1, α2 & H3.10 (Supplementary Figure 4A) [21]. 2) Residues evolutionarily conserved in the JmjN domains of KDM4A-C. 3) Residues which could potentially contribute to protein-protein interactions, preferably salt bridges which could be disrupted by extreme pH changes. The three JmjN domains from human KDM4A-C share high sequence homology (~80%), and are highly conserved in all mammals. Furthermore, the sequences are rich in charged amino acids (Supplementary Figure 4A, indicated in blue and red). We hence substituted two pairs of highly conserved acidic and/or basic residues with the neutral aa alanine, and evaluated the impact on dimerization of the full-length KDM4C and of its isolated JmjN domain. We used a two-step site-directed mutagenesis (SDM) procedure in order to introduce the two double mutations pairs, p.E24A/E25A and p.H42A/R43A, into YN- and YC-tagged KDM4C (resulting in a double mutant termed dmKDM4C), and into its YN- and YC-tagged JmjN domain (termed dmJmjN). The dmKDM4C-YN/YC plasmids were co-transfected into HEK293 cells, and IF was performed (Figure 8A). An anti-KDM4C antibody was used to detect positively transfected cells (Figure 8A, blue fluorescence) and H3K9me3 staining served as the demethylation activity readout (Figure 8A, red fluorescence). The dmKDM4C lost its homodimerization capacity as evidenced by the lack of YFP fluorescence in the anti-KDM4C-positive cells (Figure 8A, compare image e to image f). Moreover, dmKDM4C failed to erase the H3K9me3 mark, as reflected by the equivalent H3K9me3 staining in transfected and untransfected cells (Figure 8A-g). The expression of both monomers was validated by WB, to ensure that their dimerization was not affected by impaired expression (Figure 3C, lane 4). To further pin-point the minimal peptide sequence required for KDM4C dimerization, we assessed the impact of each mutation pair alone (i.e. p.E24A/E25A or p.H42A/R43A, Figure 8A, third and fourth rows, respectively). Each mutated pair significantly reduced KDM4C dimerization and H3K9me3 demethylation, at a level comparable to the effect observed with the double-mutations, although the impact of the p.H42A/R43A seemed more deleterious than that of p.E24A/E25A (Figure 8A, compare i and m to e, and, k and o to g).

In concordance with the full-length KDM4C protein, dmJmjN also displayed impaired dimerization relative to the WT JmjN, as evident from the reduced YFP fluorescence in a live cell imaging assay (Supplementary Figure 5A, compare image c to image a). This strengthened the hypothesis that the mutations directly interfere with JmjN-JmjN interactions, rather than interrupt with the fold of the JmjN domain in the context of the whole enzyme. Each mutation pair, on its own, had a less significant impact on dimerization of the isolated JmjN peptides than the double mutation, since the transfected cells exhibited relatively higher YFP fluorescence (Supplementary Figure 5A, compare images e, g to c). Notably, the introduction of the p.E24A/E25A mutation in the isolated JmjN-YN/YC monomers resulted in an expression pattern resembling mitochondrial localization (Supplementary Figure 5A-e, arrow). This was verified by co-localization of the YFP fluorescence signal with the established mitochondrial marker MitoTracker red (Supplementary Figure 5B- c and f). This new mitochondrial localization could possibly be a result of the removal of the negative charge at the N-terminus of the peptide, which now resembles a mitochondrial leader sequence [41]. The fact that the mitochondrial localization was detected indicates that the p.E24A/E25A mutation did not significantly interfere with JmjN dimerization. dmJmjN monomers of KDM4C were further visualized by IF microscopy with an anti-YFP antibody to confirm their expression (Figure 8B). While anti-YFP staining was detected, YFP fluorescence was significantly reduced in dmJmjN-expressing cells when compared to WT JmjN (Figure 8B, compare images d and a). Notably, the anti-YFP staining was also weaker in the dmJmjN-expressing cells relative to WT JmjN expressing cells (Figure 8B, compare image e to image b), probably due to incomplete folding of the YN and YC halves [42] which might lead to impaired recognition by the antibody.

### The JmjN peptide is an inhibitor of KDM4A/C demethylation activity

**Figure 9:**
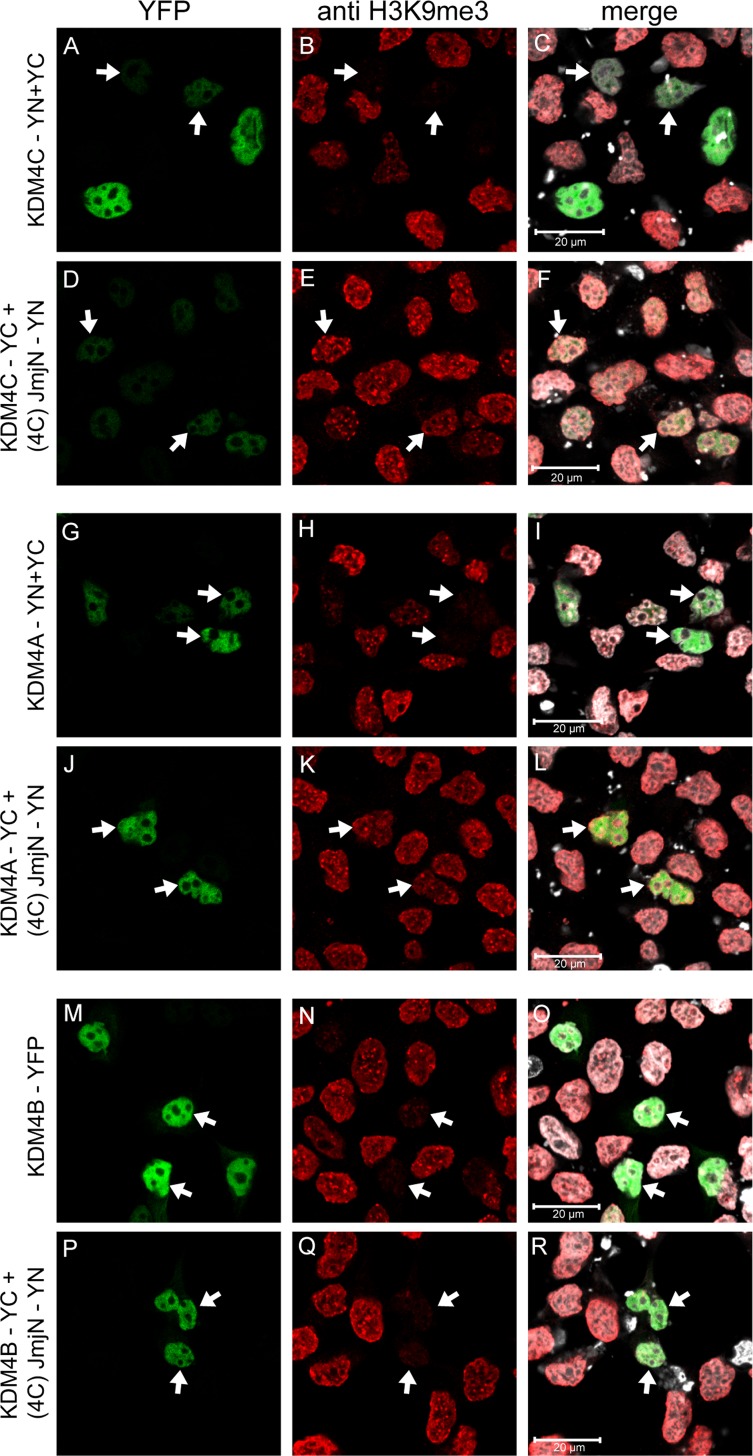
IF microscopy depicting the effect of heterodimerization with the individual JmjN peptide on H3K9me3 demethylation by KDM4A-C HEK293 cells were transiently co-transfected with the YN-tagged JmjN peptide of KDM4C [i.e. (4C)-JmjN-YN] and either of the YC-tagged KDM4A-C. The controls were either co-transfections of the YN- and YC-tagged KDM4A/C (for KDM4A/C-JmjN heterodimers) or a transfection with the YFP-tagged KDM4B (for KDM4B-JmjN heterodimer). Green fluorescence represents YFP, red fluorescence represents H3K9me3 staining, Blue fluorescence represents anti-HA staining, whereas white fluorescence represents the DNA dye DRAQ5. Arrows indicate specific cells which display dimerization of the exogenous proteins. Cells were visualized by scanning confocal microscopy at a ×63 magnification. All fields are representative of at least three independent experiments.

Our results demonstrated that dimerization of KDM4C via its JmjN domain is essential for its demethylase activity, whereas KDM4B did not dimerize efficiently suggesting that KDMB does not require dimerization for its catalytic activity. To corroborate these findings, we explored the possible dimerization of YC-tagged KDM4A-C with the individual YN-tagged JmjN peptide of KDM4C [i.e. (4C)-JmjN], and its impact on H3K9me3 demethylation (Figure 9). Dimerization with the JmjN domain, which is further stabilized by the reconstitution of the YFP tag, should prevent the assembly of any homo- or heterodimer between the full-length proteins by blocking the dimerization interface, and allow the assessment of the catalytic activity of KDM4A-C monomers. We therefore compared the demethylase activity of KDM4-JmjN heterodimers to that of the corresponding homodimers (i.e. KDM4C-YN/YC and KDM4A-YN/YC) or to the KDM4B-YFP protein, as the latter does not form homodimers and thus cannot be detected by YN/YC reconstitution. All three full-length proteins exhibited JmjN binding capacity as indicated by the assembly and fluorescence of the YFP tag (Figure 9, images D, J and P). Heterodimers of JmjN with either KDM4A or KDM4C exhibited low to complete loss of H3K9me3 demethylase activity as evident by the retention of the red fluorescence of H3K9me3 (Figure 9, arrows, compare E and K to B and H, respectively). In contrast, JmjN-KDM4B heterodimers displayed comparable H3K9me3 demethylase activity as the YFP-tagged KDM4B (Figure 9, arrows, compare Q to N). Notably, the intensity of the YFP fluorescence was weaker in the KDM4C-JmjN heterodimers than in KDM4C homodimers, suggesting that the conformation of the KDM4C-JmjN heterodimer was not compatible with optimal fluorescence complementation. However, the reduction in the catalytic activity of JmjN-KDM4C heterodimers was significant compared to KDM4C homodimers with comparably low YFP levels, which completely erased the H3K9me3 mark (Figure 9, arrows, compare D to A, and E to B). These results suggest that like KDM4C, KDM4A also requires dimerization for its demethylase activity, while KDM4B is capable of catalyzing H3K9me3 demethylation as a monomer.

## DISCUSSION

In the current study, we applied the BiFC technique to assess the *in situ* dimerization of the human histone lysine demethylases KDM4A-C in live cells. We provide a line of experimental evidence establishing the dimerization of KDM4A and KDM4C via their JmjN domain, and further show that this dimerization is absolutely required for their demethylase activity. Finally, we show that the JmjN peptide has the ability to abolish KDM4A/C dimerization and consequent demethylase activity, thus highlighting its possible application as a new cancer chemotherapeutic agent.

The *in situ* expression and dimerization of YN- and YC-tagged KDM4A and KDM4C were corroborated by live cell imaging as well as WB analyses, while their H3K9me3 demethylation activity was followed by IF microscopy, hence linking the dimerization with the demethylase activity. The JmjN domain was found as both essential and sufficient for dimerization, since its deletion in KDM4C abolished the enzyme's dimerization capacity, while the individual JmjN peptide formed homodimers. In contrast, deletion of the catalytic JmjC domain in KDM4C did not hinder its dimerization capability, thereby demonstrating that dimerization occurs independently of the catalytic activity. Furthermore, the individual JmjC peptide neither dimerized nor formed interactions with the JmjN peptide, indicating that specific JmjN-JmjN interactions facilitate dimer formation.

Apart from abrogating KDM4C's dimerization capability, we show that deletion of the JmjN domain also abolished the enzyme's demethylation activity in a manner comparable to deletion of the catalytic domain, JmjC. This has been previously reported for both the murine KDM4B [18] as well as the human KDM4A and KDM4C [19–21]. Our IF microscopy analysis shows that while deletion of either the JmjN or the JmjC domains eliminated any demethylation activity, it did not affect the histone binding capability of KDM4C, as introduction of either exogenous ΔN-KDM4C or ΔC-KDM4C resulted in an increase in nuclear H3K9me3 levels. This is likely due to saturation of KDM4C's chromatin binding sites by the catalytically dead deletion mutants, thereby blocking the access of the endogenous enzyme, resulting in higher H3K9me3 levels. Taking together the fact that both of these deletion mutants retained their histone binding capacity, nuclear localization and relatively normal expression levels, along with their opposite effects on KDM4C's dimerization capacity, one can assume that these deletions did not impair the proper overall fold of the protein. Moreover, this was the first indication that KDM4C depends on dimerization for its catalytic activity. However, since deletion of the entire JmjN domain may possibly affect the local protein structure, indirectly interrupting dimerization and activity, we introduced specific point mutations within this domain. Specifically, introduction of the mutations p.E24A/E25A and p.H42A/R43A together or independently, was sufficient to abolish the ability of KDM4C to dimerize as well as its demethylation activity. This finding that substitution of only two residues within KDM4C (containing 1056 aa) far outside the catalytic core, completely abolished both its homodimerization and demethylase activity, supports the conclusion that dimerization is crucial for KDM4C's demethylation activity. The fact that each mutation pair, on its own, was not sufficient to impair dimerization of the individual JmjN peptide, may be explained by its larger exposure area and flexibility when it is isolated from the full protein; this could allow the formation of additional bonds that are not possible when the JmjN is anchored.

Pin-pointing specific residues within the JmjN domain which may contribute to its dimerization is important for the characterization of the dimer interface and might shed light on the functional linkage between dimerization and demethylase activity. In the present study we focused on charged residues within the JmjN domain and revealed the role of possible electrostatic interactions involving aa E24/E25 and H42/R43. These residues are highly conserved within all vertebrates, suggesting their importance for dimerization. In concordance, physiological KDM4C dimers were dissociated into monomers by extreme pH conditions, suggesting the involvement of charged residues which connect through salt bridges. However, the JmjN sequence also contains a hydrophobic patch in the middle of its main helix (Supplementary Figure 4A, green line) that could be important for the stabilization of the JmjN-JmjN interaction, including two tyrosine residues which are completely conserved from human to fruit fly (Y32/Y35, Supplementary Figure 4) with a potential to form aromatic π-π interactions [43]. This was corroborated by the weakening of the physiological dimer structure upon incubation with urea. We therefore suggest a plausible dimerization mechanism in which the specificity is first determined by the formation of salt-bridges involving the conserved charged residues at the opposite short helices (Supplementary Figure 4A, α1 and H3.10), which then presumably seal the hydrophobic patch in the core interface. Hydrophobic interactions favor the exclusion of water molecules, thereby stabilizing potential hydrogen bonds between the core hydrophilic residues, resulting in a stable dimer conformation. A combined dimeric interface, which contains a hydrophobic core surrounded by hydrogen bonds, was also reported for the pyrimidine nucleotide biosynthetic regulator (PyrR) dimerization domain [44], to which the JmjN domain was found to possess structural similarities [21]. This tight dimerization modality could explain the unusually durable KDM4C dimeric structure, which could withstand the harsh SDS environment throughout the SDS-PAGE analysis. In addition to the physiological KDM4C dimers detected by WB, our findings suggest that KDM4C might also form tetramers within the nucleus, as shown by size exclusion fractionation. Albeit, one cannot determine whether all four KDM4C monomers (or possibly KDM4A) interact via the JmjN interface, or if two KDM4C dimers interact via a separate interface. However, the latter modality (i.e. dimer of dimers) seems more probable considering that KDM4C dimers were detected both in the nucleus and in the cytosol, indicating a stable conformation rather than an intermediate state. Notably, the nuclear confinement of the tetramers suggests that their assembly might be dependent on their nuclear function, or rather mediated by their association with chromatin. Interestingly, KDM4A/C oligomers might serve as a platform for simultaneous dinucleosome binding as was recently described for heterochromatin protein 1 (HP1) [45].

The final piece of evidence for the absolute requirement of dimerization for the catalytic activity of KDM4C and KDM4A was achieved without introducing any changes in the protein sequence (i.e. no deletion or aa substitution). Co-expression of either KDM4A or KDM4C with an individual JmjN domain resulted in their hetero-dimerization, thus preventing the formation of full-length enzyme homodimers. Consequently, both heterodimers of JmjN-KDM4A as well as JmjN-KDM4C displayed a marked loss of their H3K9me3 demethylase activity, thus highlighting their requirement for dimerization and that the mere interaction with a free JmjN peptide is not sufficient to acquire the demethylase activity. A plausible underlying mechanism could be that upon dimerization, the JmjC domain of KDM4A/C undergoes a crucial conformational change, involving residues outside the JmjN domain, which is required for the acquisition of demethylase activity. This concept of a bio-activating conformational change was previously suggested [21]. Importantly, KDM4B's demethylase activity was not affected by the interactions with the JmjN peptide, thus indicating that KDM4B does not require dimerization for its catalytic activity.

Quantitative image analysis of the exogenous KDM4A-C dimers revealed marked differences between the dimerization capacities of KDM4B and KDM4A/C. While KDM4A presented the highest dimerization levels, with somewhat lower capacity for KDM4C (~80%, *P-value* = 0.0009), KDM4B displayed only residual dimerization capability (~14% of KDM4A, *P-value* = 5.4 × 10^−34^). Considering the significance of dimerization to the functionality of KDM4C and KDM4A, the high homology between the three JmjN sequences and the dimerization capability of KDM4B's isolated JmjN domain, it seems surprising that KDM4B exhibited poor dimerization capacity. A possible explanation is that the JmjN domain is not as exposed in the tertiary structure of KDM4B as in KDM4A and KDM4C. This could also explain how the small isolated JmjN peptide was able to dimerize with KDM4B while the large KDM4A-C proteins were not. If the JmjN is an internal domain in KDM4B, then loss of KDM4B's catalytic activity following deletion of its JmjN domain [18] could be a result of impaired folding. In agreement with this hypothesis, when testing the activity of YFP-tagged ΔN-KDM4B in IF assays, it displayed diffused expression throughout the cell and aggregated, suggesting it underwent degradation (Supplementary Figure 2B-d). A similar effect was observed for KDM4B with the p.E23A/E24A and p.H41A/R42A mutations (i.e. dmKDM4B, Supplementary Figure 2B-g). While we show that KDM4B neither forms homodimers nor heterodimers with KDM4A/C, the study by Shin and Janknecht showed that KDM4D co-immunoprecipitated with itself but not with KDM4A/C [20]. These results suggest that interactions via the JmjN domain are distinct between different JmjN-containing proteins, while the stringency could be determined by individual residues within the sequence of each JmjN, or more likely by the extent of its water exposure. Unlike KDM4A and KDM4C, KDM4B and KDM4D were shown to be recruited to DNA damage sites and facilitate DNA double strand break repair [46, 47]. This may involve interactions of KDM4B with other proteins outside the KDM4 family, hence explaining its different dimerization requirements. In this vein, it is interesting to expand this line of investigation with other KDM4 family members, as well as explore possible interactions between KDM4A/C and other histone binding proteins, that is, exploring the putative KDM4 interactome.

The key role of dimerization shown here suggests a new level of regulation of the demethylase activity of the KDM4 enzyme subfamily. The existence of such a regulatory mechanism is expected considering the apparent redundancy of lysine-specific demethylases targeting H3K9me3 and the plethora of genomic loci reported to be occupied by KDM4 family members [48, 49]. A putative regulation mechanism might include, for instance, the control of cellular levels of KDM4A on the function of KDM4C in cases where KDM4A/C heterodimerization is required. In this respect, studies are warranted to assess whether or not heterodimerization of KDM4A-KDM4C alters substrate or chromatin loci specificity when compared to the substrates recognized by homodimers of either KDM4A or KDM4C.

Apart from enhancing our understanding of the function of KDM4 subfamily members, the current findings suggest a potential new approach for specific inhibition of KDM4A/C activity. Recent studies reported deleterious effects upon inhibition of KDM4 subfamily members in cancer cells [33, 35, 36]. In this respect, silencing KDM4C expression by siRNA led to abnormal mitotic cells [50], as well as inhibition of cancer cell growth [25, 51] and cell death in KYSE150 and U2OS cells [39], rendering these enzymes an attractive druggable target both for research as well as for anti-cancer therapeutics. Since the catalytic JmjC domain is present in many different histone lysine demethylases, designing specific competitive inhibitors has proven to be very challenging [22, 38, 52–54]. However, the JmjN domain is less common than the JmjC, and dimerization via the JmjN domain is distinct even within the KDM4 subfamily. Thus, targeting dimerization has the potential to serve as a more precise and targeted modality to selectively disrupt the demethylase activity of KDM4A/C. This could be achieved, for instance, by using a free JmjN peptide or a cognate peptidomimetic to compete on dimer formation of the full-length enzymes, thereby blocking their demethylation activity which was found to be crucial in various human malignancies.

In conclusion, the current study established that KDM4A and KDM4C, but not KDM4B, dimerize via their JmjN domain. This dimerization is a pre-requisite for their H3K9me3 demethylase activity, while KDM4B is active in its monomeric form. The JmjN-JmjN association is specific and highly stable ostensibly due to the combined effect of salt bridges and hydrophobic interactions, and free JmjN peptides can function as KDM4A/C inhibitors.

## MATERIALS AND METHODS

### Bimolecular fluorescence complementation (BiFC)

The BiFC method was used to visualize *in situ* dimerization of KDM4A-C and their individual domains in live cells, as previously described [55, 56]. In brief, an interaction between two proteins, each conjugated to a non-fluorescent half of the YFP sequence (i.e. YN or YC), mediates reconstitution of the YFP fluorophore upon dimerization such that YFP fluorescence is detected. In contrast, if the YN- and YC-conjugated proteins do not interact, no YFP fluorescence is detected. In the current study, cells were co-transfected with YN- and YC-tagged KDM4A-C enzymes or their independent domains, followed by live fluorescence imaging or immunofluorescence (IF) microscopy assays. Expression of the exogenous enzymes was confirmed using a polyclonal antibody against YFP, which recognizes both the YN and YC halves, whereas dimerization was determined by YFP fluorescence. The demethylase activity of the exogenous enzymes was verified using an anti-H3K9me3 antibody. Fluorescence microscopy was undertaken using a confocal *Zeiss* LSM 710 microscope (×63 magnification, Oberkochen, Germany). Image processing was performed using the *ZEN* black edition software.

### Cell culture

HEK293 and K562 cells were maintained in RPMI-1640 medium (Gibco, Life Technologies, Grand Isle, NY) containing 10% fetal bovine serum, 2 mM glutamine, 100 μg/ml penicillin and streptomycin (Biological Industries, Beit HaEmek, Israel), and kept in humidified air under 5% CO_2_ at 37°C.

### Cloning and site-directed mutagenesis

### Expression vectors

pcDNA3.1 vectors harboring the Venus YFP sequences [57] were kindly provided by Prof. I. D. Kerr (University of Nottingham, UK). YN refers to YFP residues 1–172 (β strands 1–8), YC refers to YFP residues 155–238 (β strands 8–11), whereas YFP represents the entire fluorescent YFP protein, as previously described [58]. Single site-directed mutagenesis (SDM, detailed below) was used to silently eliminate the *HindIII* site residing within the YN and YFP sequences (Table 2, primers 1–2), for use in the sub-cloning of KDM4A. Plasmids containing the human KDM4A-C ORF sequences (i.e. pCMV-HA-JmjD2A, pCMV-HA-JmjD2B and pCMV-HA-GASC1) were a gift from Prof. Kristian Helin (Addgene #24180, #24181 and #24214, respectively) [39]. Multiple SDM were performed on the pCMV-HA-JmjD2B plasmid, to reverse two mutations in the JmjD2B sequence (S598P and E738K; Table 2, primers 3–6) using the Change It kit according to the instructions of the manufacturer (Affymetrix, Santa Clara, CA, USA).

**Table 2:** Site-directed mutagenesis and cloning primers for KDM4A-C

#	Primer	Sequence
1	YN-no HindIII F	CAAGCTGACCCTGAA**A**CTTATCTGCACCACC
2	YN-no HindIII R	GGTGGTGCAGATAAG**T**TTCAGGGTCAGCTTG
3	4B S598P fix F	Phos-GAGGGGCAGGCACCG**T**CCACATTTTCC
4	4B S598P fix R	Phos-GGAAAATGTGG**A**CGGTGCCTGCCCCTC
5	4B E738K fix F	Phos-CCTCTGGCGGT**G**AGAACACGGAGCCGC
6	4B E738K fix R	Phos-GCGGCTCCGTGTTCT**C**ACCGCCAGAGG
7	Blunt-KDM4A	**ATATAAGCTT**GCTCCGAATTCGCCCTT
8	KDM4A-EcoRV	**TATAGATATCT**CTCCATGATGGCCCGGT
9	Blunt-KDM4B	**ATATAAGCTT**TCCGGTACCGCCATGGG
10	KDM4B-EcoRV	**TATAGATATCT**GAAGGGGGCTCCGGGC
11	Blunt-KDM4C	**ATCCTTACTC**AATTCGCCCTTATCATGG
12	KDM4C-XhoI	**ATACTCGAG**CTGTCTCTTCTGGCACTTCT
13	4A-JmjN-XhoI	**ATACTCGAG**TCGTGGCTTCCACTCTTTTG
14	4B-JmjN-XhoI	**ATACTCGA**GCCGCGGCTTCCACTCC
15	4C-JmjN-XhoI	**ATATCTCGAG**TCTTGGCTTCCACTCCTTA
16	4C-ATG-JMJC	**CTTATCATG**GATGAGGGTGTGGATGAATG
17	4C-JmjC-XhoI	**ATATCTCGAG**GCAAGTGCACAATTTGGC
18	KDM4C-delJMJN F	GAACCCCAGCTGTAAGCAGTGCTATGATGAC
19	KDM4C-delJMJN R	GTCATCATAGCACTGCTTACAGCTGGGGTTC
20	KDM4C-delJMJC F	GCAGATATTAATGGGAGCATATATAGGAAAGACATGGTGAAG
21	KDM4C-delJMJC R	CTTCACCATGTCTTTCCTATATATGCTCCCATTAATATCTGC
22	KDM4B-delJMJN F	CCAGAACCCCAGCTGTAAACAGACGTATGATGACATCG
23	KDM4B-delJMJN R	CGATGTCATCATACGTCTGTTTACAGCTGGGGTTCTGG
24	KDM4C E24A/E25A F	CAGACCCTCCATGG**C**GG**C**GTTCCGGGAGTTCAAC
25	KDM4C E24A/E25A R	GTTGAACTCCCGGAAC**G**CC**G**CCATGGAGGGTCTG
26	KDM4C H42A/R43A F	GGAGTCTAAAGGAGCC**GC**T**GC**TGCGGGTCTTGCAAAGGTG
27	KDM4C H42A/R43A R	CACCTTTGCAAGACCCGCA**GC**A**GC**GGCTCCTTTAGACTCC
28	KDM4B E23A/E24A F	GACGTTTCGCCCAACCATGG**C**AG**C**ATTTAAAGACTTCAAC
29	KDM4B E23A/E24A R	GTTGAAGTCTTTAAAT**G**CT**G**CCATGGTTGGGCGAAACGTC
30	KDM4B H41A/R42A F	GAGTCGCAGGGAGCC**GC**C**GC**GGCGGGCCTGGCCAAG
31	KDM4B H41A/R42A R	CTTGGCCAGGCCCGCC**GC**G**GC**GGCTCCCTGCGACTC

Nucleotides in bold within the sequence represent the mutation which was inserted by SDM, whereas at the 5′ end they denote an overhang. Underlined nucleotides are those flanking a deletion mutation.

### Sub-cloning of the KDM4 family members and their JmjN/C domains

The ORF of KDM4A-C, their individual JmjN domains as well as the JmjC domain from KDM4C were PCR-amplified from the pCMV-HA vectors (Table 2, primers 7–12, 7 + 13, 9 + 14, 11 + 15 and 16 + 17, respectively) using the Q5 High-Fidelity DNA Polymerase, according to the instructions of the manufacturer (New England BioLabs, Ipswich, MA, USA) and purified with the Wizard PCR & gel cleanup kit (Promega, Fitchburg, WI, USA). The JmjN domains were cloned from the first methionine and consist of aa 1–56 in KDM4A, 1–57 in KDM4B and 1–58 in KDM4C, while the JmjC domain of KDM4C consists of aa 144–310.

Restriction enzymes were purchased from New England BioLabs, and ligation procedures were performed using the DNA ligation kit 2.1 according to the instructions of the manufacturer (Takara, Otsu, Shiga, Japan). All chimeric genes harbor the YFP sequences at the C-terminus. The KDM4A sequence was ligated into the YC vector following *EcoRV* digestion, and then sub-cloned into the YN and YFP vectors using *HindIII-EcoRV* digestion. KDM4B and KDM4C were sub-cloned into the YC, YN and YFP vectors using *EcoRV* and *EcoRV-XhoI* digestion, respectively. The JmjN domains from KDM4A-C and the JmjC domain from KDM4C were inserted into YC, YN and YFP vectors using *EcoRV-XhoI* digestion.

Point mutations and deletions were introduced into the sequences of KDM4B and KDM4C by single SDM procedures (Table 2, primers 18–29), using the *pfuUltra* high fidelity DNA polymerase (Agilent, Santa Clara, CA, USA) followed by 1 h digestion with *DpnI* (Fermentas, Waltham, MA, USA) and transformation. Deletion of the JmjN domain from KDM4C includes aa 16–58, while deletion of the JmjC domain spans aa 144–310. In KDM4B, deletion of the JmjN domain includes aa 15–57.

All vectors were transformed into heat-shock DH5α *E. coli*, which were seeded on LB-agar plates containing 100 mg/L ampicillin (Sigma Aldrich, St. Louis, MO, USA). Plasmids were isolated using the GeneJET plasmid miniprep kit (Thermo Fisher Scientific, Waltham, MA, USA) and sequenced at Hylabs laboratories (Rehovot, Israel).

### Transient transfections

For IF microscopy, 4 × 10^4^ HEK293 cells were seeded 48 h prior to transfections in 24-well plates on sterile glass coverslips, in 0.5 ml growth medium per well. For live imaging, cells were seeded in 24-well glass bottom plates (Cellvis, Mountain View, CA, USA). Transfections with the various expression vectors (1 μg plasmid DNA) were performed using linear polyethylenimine (PEI, MW 25,000) transfection reagent (Polysciences, Warrington, PA, USA) at a ratio of 3 μg PEI: 1 μg plasmid DNA. For Western blots (WB), 4.8 × 10^5^ cells were seeded 48 h prior to transfections in 60 mm plates, in 5 ml growth medium per plate. Transfections were similarly performed, with a total of 5 μg of the expression vectors. In order to ensure equal amounts of vector DNA in all the transfections, an empty pcDNA3.1 vector was added in transfections of a single plasmid.

### Immunofluorescence and live imaging

IF microscopy assays were performed 20 h after transfections as follows: cells were washed twice with PBS, fixed with fresh solution of 4% formaldehyde in PBS for 15 min and washed twice for 5 min with PBS. Permeabilization was then performed using 0.1% Triton X-100 in PBS for 10 min followed by two washes with PBS. Cells were incubated for 1 h at room temperature (RT) in TBS buffer (10 mM Tris, 150 mM NaCl, pH 7.4) containing 20% skim milk, and then incubated with primary antibodies (Table 3) for 1 h at RT. Following three 5 min washes with PBS, cells were co-incubated with fluorescent secondary antibodies (Table 3) along with either 1 μg/ml Hoechst 33342 (Sigma Aldrich) or 5 μM DRAQ5 (Abcam, Cambridge, UK), for 1 h at RT in the dark. Cells were then washed three times with PBS for 5 min and the coverslips were mounted onto microscope slides over fluoromount-G (Thermo Fisher Scientific). Fluorescence was recorded using a confocal *Zeiss* LSM 710 microscope (×63 magnification), using the same laser intensities and detector gain for all slides within the experiment. For live imaging, 20 h after transfections, cells were washed with growth medium and incubated in growth medium supplemented with 1 μg/ml Hoechst 33342. For mitochondrial staining, cells were incubated with 100 nM MitoTracker red (Thermo Fischer Scientific) for 30 min prior to microscope imaging. Fluorescence was recorded using a confocal *Zeiss* LSM 710 microscope (×63 magnification), during incubation at 37°C in an atmosphere of 5% CO_2_. Image processing was performed using the *ZEN* Black edition software.

**Table 3:** Antibodies used in Western blot and immunofluorescence microscopy assays

Antibody	Company	WB	IF	Catalog no.
Rabbit polyclonal anti-YFP	*Medical and Biological Laboratories (Woburn, MA, USA)*	1:2000	1:1000	598
Mouse monoclonal anti-KDM4C	*Santa Cruz (Dallas, TX, USA)*	1:500	1:400	sc-515767
Rabbit polyclonal anti-KDM4D	*Active motif (Carlsbad, CA, USA)*	1:1000	–	39247
Rabbit polyclonal anti-H3K9me3	*Abcam (Cambridge, UK)*	−	1:500	ab8898
Goat polyclonal anti-HA tag	*Santa Cruz (Dallas, TX, USA)*	−	1:200	sc-805
Rhodamine Red-X-conjugated affinipure donkey anti-rabbit IgG	*Jackson Immunoresearch (West Grove, PA, USA)*	−	1:100	711-295-152
DyLight 405-conjugated affinipure goat anti-mouse IgG	*Jackson Immunoresearch (West Grove, PA, USA)*	−	1:400	115-475-062
DyLight 405-conjugated affinipure donkey anti-goat IgG	*Jackson Immunoresearch (West Grove, PA, USA)*	−	1:200	705-475-147
HRP-conjugated affinipure goat anti-rabbit IgG	*Jackson Immunoresearch (West Grove, PA, USA)*	1:10000	−	111-035-045
HRP-conjugated affinipure goat anti-mouse IgG	*Jackson Immunoresearch (West Grove, PA, USA)*	1:15000	−	115-035-062

### Quantitative image analysis

For the quantification of the extent of KDM4A-C dimerization we performed IF microscopy, as detailed above, following transfection with the various YN- and YC-tagged expression vectors. Cells positive for transfection were detected using an anti-YFP antibody, while dimerization was detected by the reconstitution of the YFP tag. For each transfection we recorded ~15 fields (such that there were >100 cells for each analysis) in 12-bit images. Utilizing the Imaris – 3D/4D/5D Image analysis software, we programmed the algorithm to recognize anti-YFP positive cells, and determined their mean YFP fluorescence intensity. We used a two-tailed heteroscedastic Student's *t*-test to examine the statistical significance of the differences in fluorescence intensity between the cells from each transfection. A difference was considered significant if the *P*-value obtained was < 0.025.

### Western blot analysis

For analysis of full-length KDM4A-C proteins, NP extracts were prepared 20 h after transfections by first isolating the nuclei from the cytosol. For this purpose, cells were incubated in a hypotonic buffer (10 mM HEPES pH 8, 10 mM KCl, 0.1 M EDTA, 0.1 M EGTA and 1 mM DTT, supplemented with cOmplete Mini, EDTA free, Roche, Basel, Switzerland) for 15 min on ice, and lysed with 0.5% NP-40. Nuclei were sedimented by centrifugation (30 sec at 20,000 × g, 4°C), and NPs were extracted by three rounds of 5 sec sonication pulses in fresh hypotonic buffer (Microson, amplitude 3, Misonix, Farmingdale, NY, USA) followed by centrifugation (15 min, 20,000 × g, 4°C). For analysis of the individual JmjN and JmjC domains, which localizes equally in the nucleus and cytosol, total cell extracts (TP) were isolated by incubating the cells on ice in the hypotonic buffer for 15 min, followed by sonication and a 15 min centrifugation at 20,000 × g at 4°C to eliminate cell debris. Protein concentration was determined using the Bio-Rad protein Assay (Bio-Rad, Hercules, CA, USA). For the analysis of the endogenous KDM4C protein from K562 cells, NPs were isolated and 70 μg protein aliquots were treated with either 4 M urea, citric acid:Na_2_HPO_4_ buffer (pH = 4) or CAPS buffer (pH = 11) at 37°C for 20 min prior to resolution by SDS-PAGE on a 4–15% gradient polyacrylamide gel (Mini-PROTEAN TGX gel, Bio-Rad). For analysis of exogenous YN- and YC-tagged proteins, 40 μg NPs or 50 μg TPs were resolved by SDS-PAGE on a 6% polyacrylamide gel with PM2800 Excel-Band 3-color Extra Range Protein Marker (SMOBIO Technology, Hsinchu, Taiwan) for full KDM4B-C proteins, or a 12.5% polyacrylamide gel with Spectra Multicolor Broad Range Protein Ladder (Thermo Fisher Scientific) for isolated JmjN and JmjC domains. The resolved proteins were electroblotted onto a Protran BA83 cellulose nitrate membrane (WhatmanTM, GE, Maidstone, UK). Membranes were blocked for 1 h at RT in TBS buffer containing 20% skim milk, reacted with primary antibodies (Table 3) and rinsed three times for 10 min with washing buffer (TBS supplemented with 0.5% Tween 20) at RT. The blots were then reacted with horseradish peroxidase (HRP)-conjugated secondary antibodies (Table 3) for 1 h at RT and rinsed three times for 10 min with washing buffer at RT. Enhanced chemiluminescence (ECL) detection was performed using the EZ-ECL kit, according to the manufacturer's instructions (Biological Industries, Beth-Haemek, Israel), and recorded by ImageQuant LAS 4000 imaging system (GE Healthcare Life Sciences, Marlborough, MA, USA). Prior to reprobbing with a loading control, the membrane was stripped in stripping buffer (0.5 M acetic acid, 0.5 M NaCl pH = 2.6) for 10 min at RT and washed twice with wash buffer.

### Size exclusion HPLC

K562 cells were harvested at the mid-log phase of growth for cytosolic protein (CP) and NP extractions in hypotonic buffer as described above. NPs (2 mg) or CPs (4 mg) were loaded onto a gel filtration Superdex 200 Increase 10/300 GL column in an AKTA AVANT chromatography system (GE healthcare Life Sciences) using the hypotonic buffer. Fractions of 0.5 ml were collected, and the protein-containing samples were concentrated using Amicon Ultra Centrifugal Filters (10 k cutoff) according to the instructions of the manufacturer (Merck, Burlington, MA, USA). The samples were then acidified to pH = 4 as described above and visualized by WB analysis with an anti-KDM4C antibody. A sample (80 μg) of the NP input was used as a positive control. The following size markers were loaded onto the column separately and their elution volumes were determined by 280 nm absorption, as previously described [59]: Thyroglobulin (669 kD), Ferritin (440 kD), Catalase (232 kD), Aldolase (158 kD), and BSA (67 kD).

### JmjN sequence alignment and conservation analysis

For comparison between the JmjN domains from human KDM4A-C, their sequences (aa 14–56, 15–57 or 16–58, respectively, based on the Uniprot database) were aligned using the National center for biotechnology information (NCBI) BLAST tool. For conservation analysis, the sequence of the JmjN domain from human KDM4C was used as query in a BLAST search against the reference proteins database, excluding models and uncultured/environmental sample sequences. Selected JmjN sequences from KDM4A-C in different organisms were presented by the NCBI multiple sequence alignment (MSA) viewer in the frequency-based difference coloring system.

## SUPPLEMENTARY MATERIALS FIGURES



## Abbreviations

aa: Amino acid
BiFC: Bimolecular fluorescence complementation
CP: cytosolic proteins
dm: Double mutant
HPLC: high pressure liquid chromatography
IF: Immunofluorescence
JMJD: Jumonji C domain-containing protein
KDM: Lysine demethylases
KMT: Lysine methyl transferase
MW: Molecular weight
NP: Nuclear protein
PHD: Plant homodomain
SDM: site-directed mutagenesis
TP: Total protein
WB: Western blot
HRP: Horseradish peroxidase
