# Supplementary material for: The JmjN domain as a dimerization interface and a targeted inhibitor of KDM4 demethylase activity
Source: Oncotarget. 2018 Mar 30;9(24):16861–82. doi: 10.18632/oncotarget.24717 (PMC5908291; doi:10.18632/oncotarget.24717)
Supplement: Supplementary file 1 [file oncotarget-09-16861-s001.pdf]

The JmjN domain as a dimerization interface and a targeted inhibitor of KDM4 demethylase activity

SUPPLEMENTARY MATERIALS

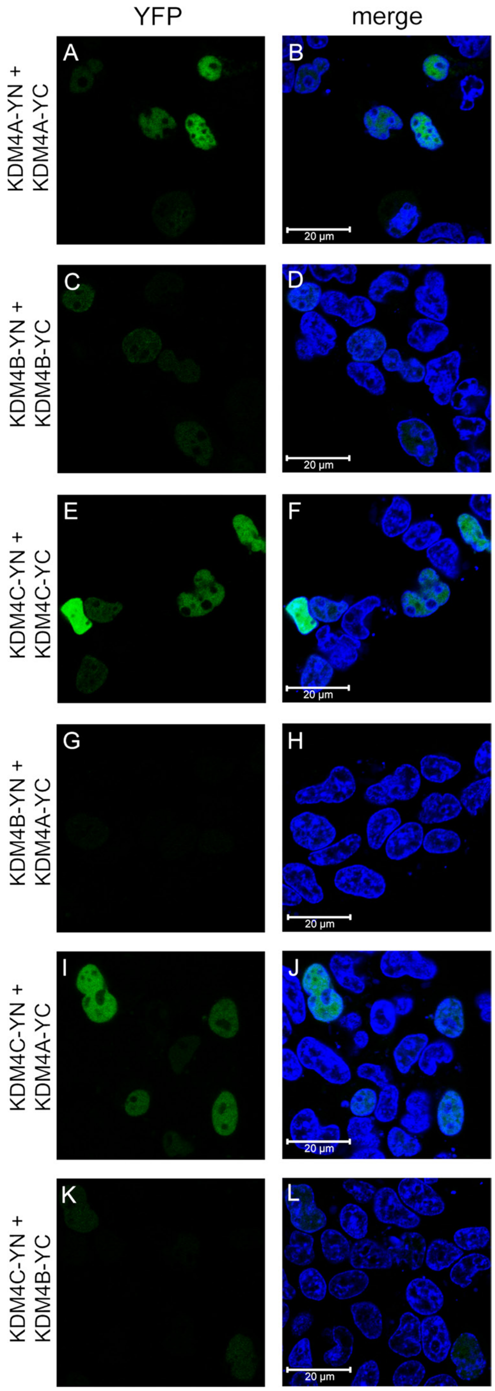

Supplementary Figure 1: Live cell imaging displaying the dimerization of YN- and YC-tagged KDM4A-C monomers. Transfected HEK293 cells harboring YN- and YC-tagged KDM4A-C were visualized by a scanning confocal microscope at a ×63 magnification. Green fluorescence represents YFP that was reconstituted by the refolding of the YN- and YC halves, while blue fluorescence represents the DNA dye Hoechst 33342. All fields are representative of at least three independent experiments.

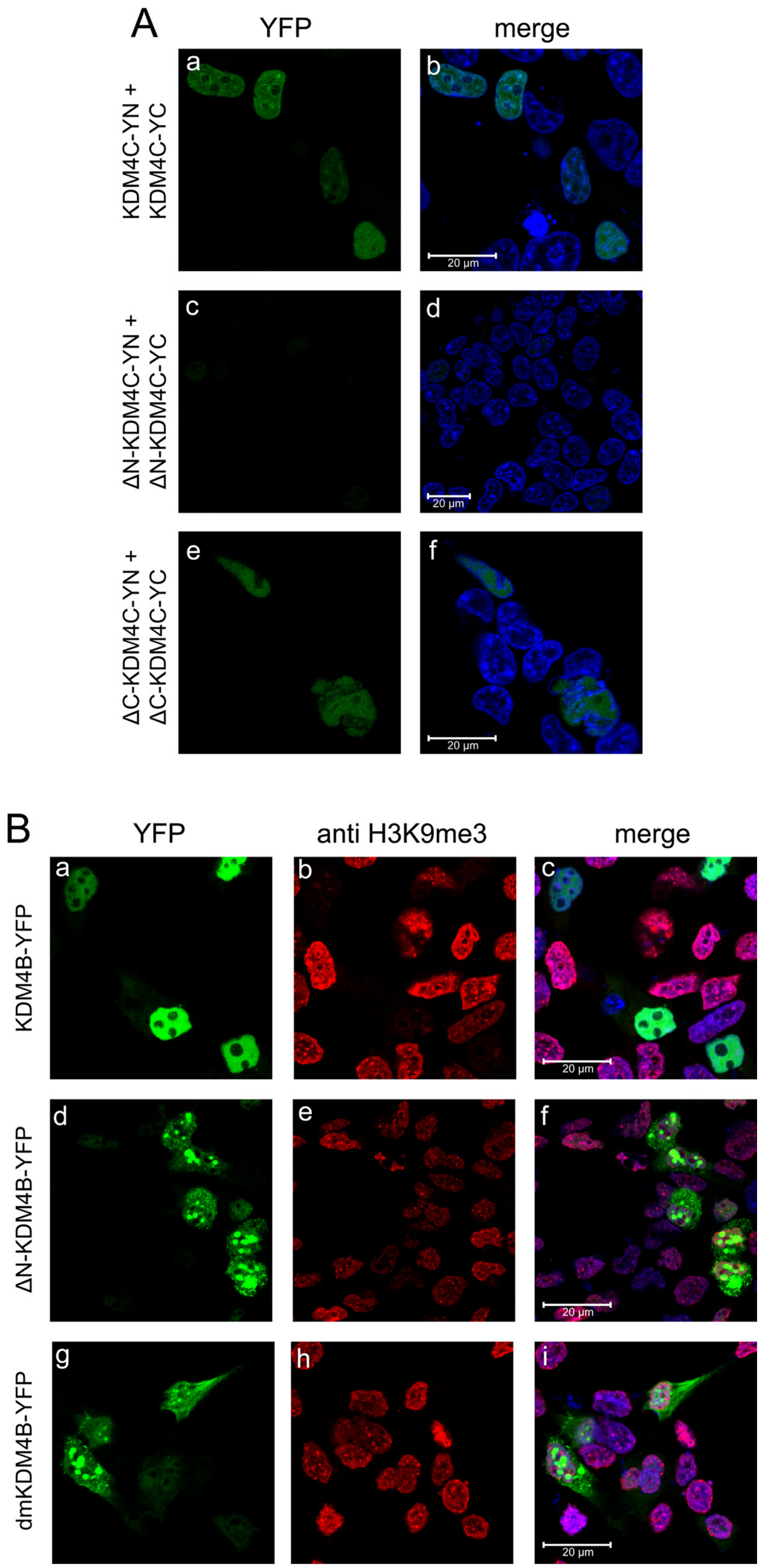

Supplementary Figure 2: Confocal microscopy exploring the effect of JmjN/C deletions on the expression and dimerization of KDM4C and KDM4B. HEK293 cells were either co-transfected with YN- and YC-tagged KDM4C with deletion of the JmjN or the JmjC domains and visualized by live cell imaging (**A**), or transfected with YFP-tagged JmjN-deleted KDM4B or with double-mutated p.E23A/E24A + p.H41A/R42A KDM4B (termed dmKDM4B) and visualized by IF microscopy (**B**). Green fluorescence represents YFP that was reconstituted by the refolding of the YN and YC halves (A) or the full YFP tag (B), blue fluorescence represents the DNA dye Hoechst 33342, while red fluorescence represents H3K9me3 staining. Cells were visualized by a scanning confocal microscopy at a x63 magnification. All fields are representative of at least three independent experiments.

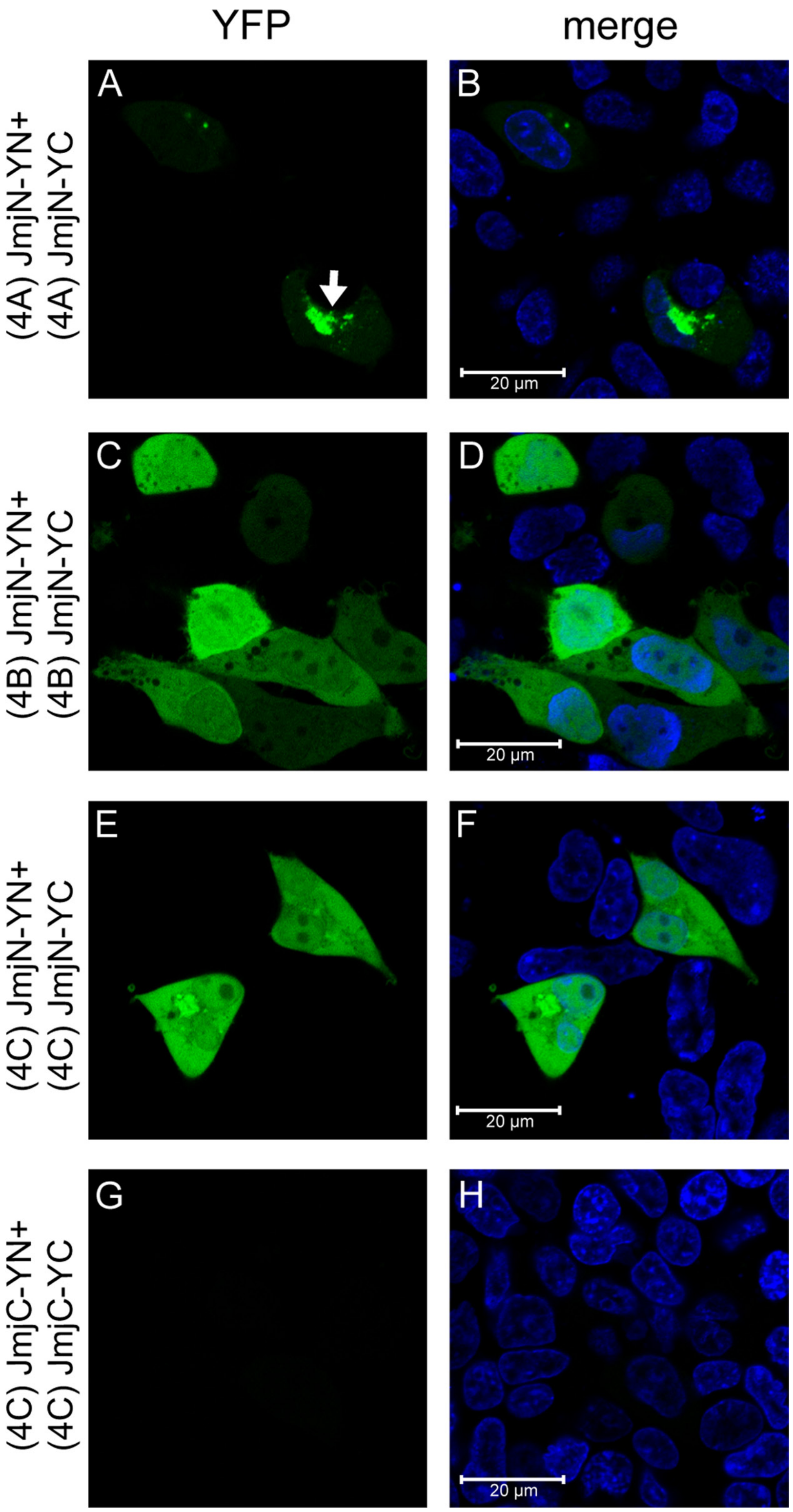

Supplementary Figure 3: Live cell imaging exploring dimerization of the independent JmjN and JmjC domains. HEK293 cells were transfected with expression vectors harboring YN and YC-conjugates of JmjN sequences from KDM4A-C, or the JmjC sequence from KDM4C, and visualized by live cell imaging. The arrow points to aggregation of JmjN dimers from KDM4A. Green fluorescence represents YFP that was reconstituted by the refolding of the YN and YC halves and blue fluorescence represents the DNA dye Hoechst 33342. Cells were visualized by a scanning confocal microscope at a ×63 magnification. All fields are representative of three independent experiments.

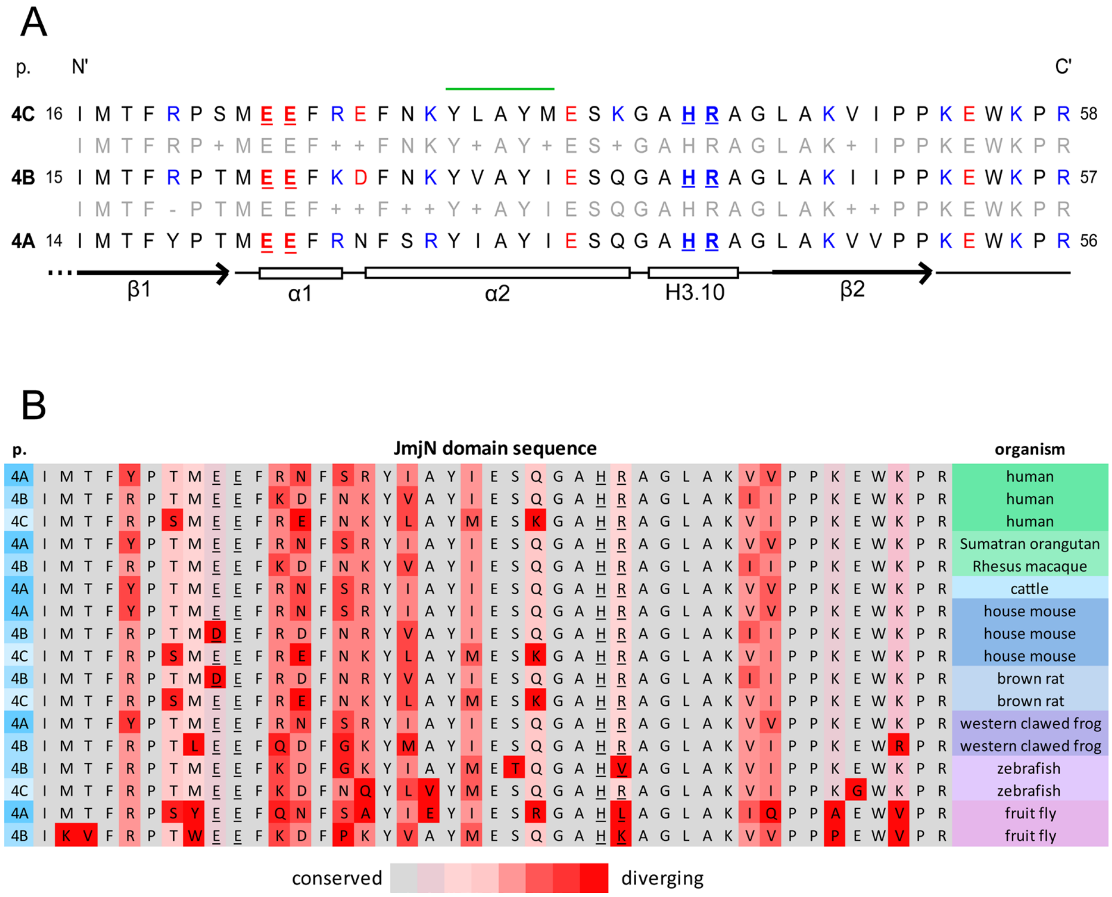

Supplementary Figure 4: Alignment of the JmjN domain sequences from KDM4A-C. (**A**) human JmjN peptide sequences from KDM4A-C (i.e. 4A–4C), as defined in the Uniprot web page, were aligned using the NCBI BLAST tool. Acidic residues are colored in red while basic residues are colored in blue. Residues that were substituted with alanine by SDM are underlined, whereas the green line marks the hydrophobic patch in the middle of the sequence. The secondary structure indicated at the bottom is based on the crystal structure of KDM4A [21]. Plus signs represent alignment of different aa with similar chemical properties while the minus sign represents alignment of aa with contrasting chemical properties. The numbers indicate the position of the sequences within the full-length proteins. (**B**) Conservation of the JmjN sequence from KDM4A-C in different organisms, presented in *frequency-based difference* coloring system. The color represents the distance of each residue from the consensus in its column, light grey being the most conserved and bright red the most divergent. Residues that were substituted with alanine by SDM are underlined.

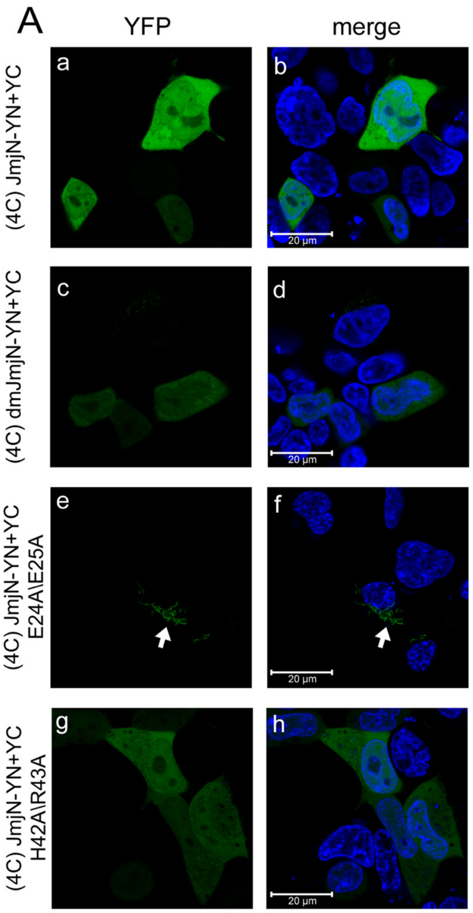

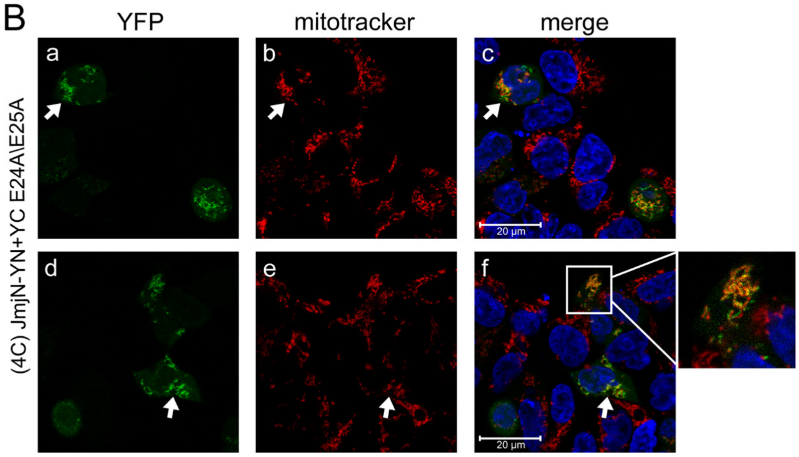

Supplementary Figure 5: The effect of p.E24A/E25A and p.H42A/R43A mutations on the expression and dimerization of the individual JmjN domain from KDM4C. HEK293 cells were transfected with expression vectors harboring YN and YC-conjugates of WT or mutant JmjN sequences from KDM4C, and visualized by live cell imaging. (**A**) Expression and dimerization of mutant JmjN harboring either one or both p.E24A/E25A and p.H42A/R43A mutations (i.e. double mutant, dmJmjN). The arrow points to mitochondrial YFP staining. (**B**) Expression, dimerization and mitochondrial localization of p.E24A/E25A JmjN dimers. Cells were stained with the mitochondrial marker MitoTracker red. The inset shows a magnified (4×) view of the box in the merged image. Green fluorescence represents YFP that was reconstituted by the refolding of the YN and YC halves, blue fluorescence represents the DNA dye Hoechst 33342 and red fluorescence denotes the mitochondrial dye MitoTracker-red. Cells were visualized by a scanning confocal microscope at a ×63 magnification. All fields are representative of three independent experiments.
